# Lattice-free prediction of three-dimensional structure of programmed DNA assemblies

**DOI:** 10.1038/ncomms6578

**Published:** 2014-12-03

**Authors:** Keyao Pan, Do-Nyun Kim, Fei Zhang, Matthew R. Adendorff, Hao Yan, Mark Bathe

**Affiliations:** 1 Department of Biological Engineering, Massachusetts Institute of Technology, 77 Massachusetts Avenue, Building 16, Room 255, Cambridge, Massachusetts 02139, USA; 2 Department of Mechanical and Aerospace Engineering, Seoul National University, 301-dong 1516-ho, Gwanak-ro 1, Gwanak-gu, Seoul 151-744, Republic of Korea; 3 Center for Molecular Design and Biomimicry, the Biodesign Institute, Arizona State University, Tempe, Arizona 85287, USA; 4 Department of Chemistry and Biochemistry, Arizona State University, Tempe, Arizona 85287, USA

## Abstract

DNA can be programmed to self-assemble into high molecular weight 3D assemblies with precise nanometer-scale structural features. Although numerous sequence design strategies exist to realize these assemblies in solution, there is currently no computational framework to predict their 3D structures on the basis of programmed underlying multi-way junction topologies constrained by DNA duplexes. Here, we introduce such an approach and apply it to assemblies designed using the canonical immobile four-way junction. The procedure is used to predict the 3D structure of high molecular weight planar and spherical ring-like origami objects, a tile-based sheet-like ribbon, and a 3D crystalline tensegrity motif, in quantitative agreement with experiments. Our framework provides a new approach to predict programmed nucleic acid 3D structure on the basis of prescribed secondary structure motifs, with possible application to the design of such assemblies for use in biomolecular and materials science.

## Abstract (web summary)

DNA may be used to fabricate functional nanostructures with various possible geometries, but first being able to predict these structures is a challenging task. Here, the authors use coarse-grained modelling to predict the shape of artificial DNA nanostructures in solution.

## Graphical abstract

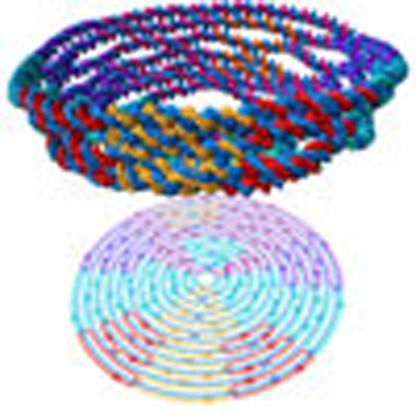

Sequence-design principles for programming nucleic acids to self-assemble into stable, highly structured macromolecular assemblies date back to foundational work by Ned Seeman in the early 1980s^1^. There, it was demonstrated theoretically that canonical Watson–Crick basepairing of complementary DNA strands could in principle be used to program stable DNA-based assemblies of vastly larger scale than the double helix itself. Core structural elements of this synthetic biomolecular design paradigm were conceived to be immobile multi-way junctions interconnected by double-stranded DNA (dsDNA) duplexes^2^,^3^,^4^.

Since this landmark work, a myriad of two-dimensional (2D) and three-dimensional (3D) structured nucleic acid assemblies have been designed using the foundational principles established by Seeman, exploiting a variety of topological routing and sequence-design strategies that include the highly successful scaffolded DNA origami approach^5^. In this approach, hundreds of short synthetic single-stranded nucleic acids are combined with a single, long scaffold strand that is typically the M13 phage genome to program megadalton-scale architectures. Examples include brick-like rectilinear^5^,^6^ and curved^7^ assemblies designed on square or honeycomb lattices, in which anti-parallel DNA helices are constrained to their topologically adjoined neighbours via stacked four-way junctions, generalized gridiron-like rectilinear and curved objects in 2D and 3D space^8^,^9^, as well as other examples^10^,^11^. In the absence of a scaffold, single-stranded DNA can alternatively be assembled alone to form extended 2D^12^,^13^ and 3D^14^ lattices and crystals, as well as finite-sized cage-like objects^15^,^16^,^17^.

Although a number of sequence-design paradigms exist to program the secondary structure of ordered DNA assemblies using the high affinity and specificity of DNA hybridization, there is no generalized computational procedure to predict the 3D structure of these assemblies that is central to their functions as nanoscale materials. Although atomic-based modelling approaches such as molecular dynamics (MD) are attractive in their ability to capture detailed atomic-level interactions and subtle conformational features, they are limited to the simulation of time-scales up to hundreds of nanoseconds, even for moderately sized assemblies, whereas DNA origami folding times typically span hours to days.

For this reason, we previously introduced the coarse-grained finite element model CanDo to predict 3D solution structure efficiently^18^,^19^. In this approach, B-form DNA is treated as a mechanical element with empirical stretching, bending and torsional stiffness, which was shown to be crucial to accurately predict the 3D structure of complex nucleic-acid architectures in which nearest-neighbour DNA duplexes are constrained on parallel square or honeycomb lattices^18^,^19^. Although powerful for such lattice-based structures that are typically designed using the sequence-design software caDNAno^20^, in which neighbouring DNA duplexes reside in an antiparallel arrangement, our framework could not be applied more generally to model DNA architectures consisting of multi-way junctions constrained by DNA duplexes in 3D space. Moreover, the framework could not treat closed-DNA topologies such as rings- and cage-like objects, further limiting its scope^18^,^19^,^21^.

To overcome these limitations and enable the prediction of the 3D structure of high molecular weight-programmed DNA assemblies based on their underlying sequence topology, here we develop a junction-centric structure-prediction framework that parses programmed secondary structure motifs to model nucleic acid assemblies consisting of arbitrary connections of multi-way junctions interconnected by DNA duplexes. We focus its application to the immobile four-way junction in the present work due to its widespread use in structural DNA nanotechnology. Although four-way junctions are known to exhibit complex and subtle structural properties depending on solvent conditions^2^,^3^,^4^ and whether they are isolated^22^,^23^,^24^ or constrained via integration into larger-scale objects^25^,^26^, we illustrate here the application of our approach assuming a single ground-state geometry in which duplexes arrange into a preferred ‘stacked-X’ configuration, with geometry and mechanical properties that are known empirically. This work lays the foundation for the future exploration of their contextually and solvent-dependent structural properties in large-scale DNA-based objects, including possibly modelling preferences for alternative isomerization states, and application of the framework to alternative multi-way junctions that may in principle also be RNA-based^27^. To demonstrate the utility of our approach, we apply it to a diverse set of DNA-based assemblies consisting of circular rings, tiles and crystalline lattices, and compare model predictions with experiments.

## Results

### Parsing programmed secondary structure

**Figure 1:**
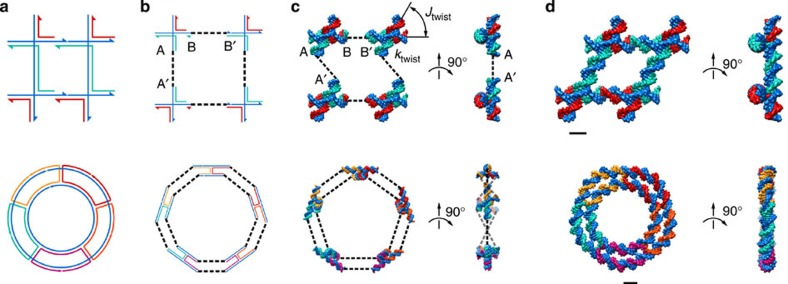
3D structure-prediction procedure for two exemplary designs. (**a**) Topological routing of DNA strands shown in different colors with arrowheads representing the 3′ ends. (**b**) The topological routing is subsequently divided into interconnected four-way junctions. (**c**) These junctions are input as the initial configuration of the finite element model generated. Connectivities between junctions are shown as dashed lines. For example, duplex end A connects A′, and B connects B′. Initial junction configurations are parameterized by a scissor-like interhelical angle *J*_twist_ and junction twist angle stiffness *k*_twist_. (**d**) Final solution structures computed from their initial, stress-free configurations. Finite element models are visualized using corresponding atomic models generated using DNA strands colored the same as in their topological representations. Scale bars are 2 nm.

Our procedure begins by parsing a sequence-based topological representation of the nucleic-acid nanostructure that can contain arbitrary secondary structural elements including duplexes and multi-way junctions. Although we use the software Tiamat here to represent nucleic-acid secondary structure^28^ and limit our treatment to stacked-X four-way junctions, our approach is equally applicable to other *N*-way junctions specified using other software. Initial nucleic-acid basepair (bp) positions and orientations are also specified in 3D space by the designer, where duplexes can be joined via four-way junctions in arbitrary-specified topologies rather than constrained to lie in an antiparallel orientation along honeycomb or square lattices^6^,^7^. Although this offers great flexibility in modelling complex DNA nanostructure geometries, stacked-X four-way junctions must be specified to remain in one of two possible isomeric states because their inter-conversions are not presently modelled. As representative toy examples, consider the 2 × 2 four-way junction tile originally introduced by Mao and Seeman^24^ and a simple two-layer ring (Fig. 1). The programmed secondary structure sequence designs are first interpreted as directed graphs, from which all four-way junctions together with their isomeric states and connecting duplex arms are identified, including nucleotide identities and their topological connectivity in nucleic-acid strands (Supplementary Figs 1 and 2 and Supplementary Note 1).

### Predicting 3D structure

To compute the equilibrium 3D structure of each assembly, we use the finite element method to solve for the ground-state minimum energy structure that satisfies both topological constraints and empirically assumed ground-state geometries and flexibilities of duplexes and four-way junctions (Supplementary Note 2 and Supplementary Datasets 1–14). Although the finite element model requires an initial position and orientation of each duplex and four-way junction in their ground-state geometries that are chosen here to be specified by the designer, we note that this structure may in principle be far from the ground-state, mechanical equilibrium structure (Fig. 1, Supplementary Figs 3–5, and Supplementary Note 3). B-form DNA is modelled as a worm-like chain with axial rise of 0.34 nm per bp, right-handed helicity of 10.5 bp per turn^6^,^7^,^29^,^30^, stretch modulus of 1,100 pN, bend modulus of 230 pN nm^2^, and twist modulus of 460 pN nm^2^, as previously assumed^18^,^19^. Effects of nicks are explored separately by assuming a reduced mechanical stiffness of the duplex, as also assumed previously^18^,^19^. Four-way junctions are assumed to exist in a single, known ground-state ‘stacked-X’ configuration with interhelical distance that is chosen to be 1.85 nm at the junction to be consistent with atomic models^25^, a right-handed twist of 60° (refs 24, 31, 32), and a rotational stiffness of *k*_twist_=135 pN nm rad^−1^ of the scissor-like interhelical angle *J*_twist_ (Fig. 1 and Supplementary Figs 6–8)^33^. The rotational stiffness of *J*_twist_ is estimated empirically in the present work using the equilibrium distribution of *J*_twist_ from MD simulations of an isolated four-way junction (PDB ID: 1DCW)^23^, and cross-validated using published Förster resonance energy transfer measurements^4^,^34^ (Supplementary Figs 9 and 10 and Supplementary Note 4). The existence of alternative ground-state *J*_twist_ angles, multiple *J*_twist_ energy minima and nonlinear *J*_twist_ mechanical response functions are ignored together with their sequence-, solvent- and contextual-dependence in parallel^35^, anti-parallel or other designed crossover geometries. Electrostatic interactions between neighbouring duplexes are also ignored in assuming that mechanical forces and torques propagated through duplexes and junctions dominate in determining overall nanostructure shape^18^,^19^. An important conceptual advance over our previous modelling framework is that the overall equilibrium ground-state 3D structure of DNA assemblies, which may in principle contain up to hundreds or thousands of such four-way junctions interconnected through duplexes, can subsequently be computed using constraint equations that force continuous duplex ends that are initially distant in space to be topologically continuous in the final configuration (Fig. 1, Supplementary Fig. 5, Supplementary Note 3, and Supplementary Movie 1). Future extensions to this model may explore incorporating the complex sequence-, solvent- and contextual-dependence of four-way junction ground-state geometric and mechanical properties, inter-changeable four-way junction isomeric state, as well as electrostatic effects.

### Topologically closed DNA assemblies

**Figure 2:**
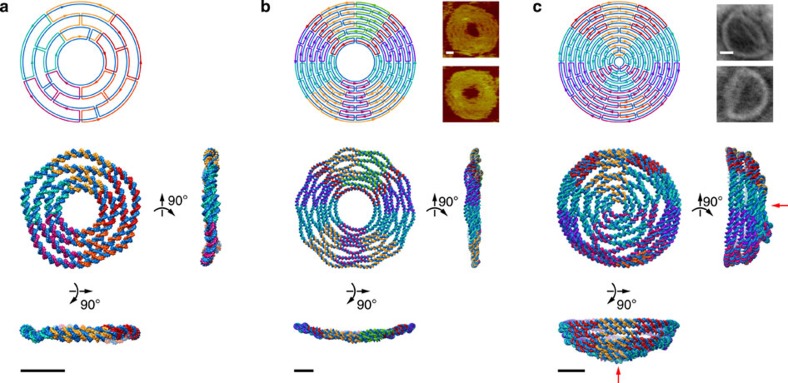
Concentric ring structures. (**a**) four-layer ring, (**b**) nine-layer ring origami and (**c**) 12-layer hemispherical origami. Each panel shows the sequence and topology design, as well as three orthogonal views of the atomic model corresponding to the final 3D structure. The AFM images of the 9-layer ring origami and transmission electron microscopy images of the 12-layer hemispherical origami are adapted from the literature^8^, reprinted with permission from AAAS. All scale bars are 10 nm. The initial configurations used for the finite element calculation are shown in Supplementary Fig. 28. The red arrows indicate the slightly collapsed upper quadrant in the 12-layer hemispherical origami.

To test the ability of our procedure to be applied to topologically closed DNA assemblies that cannot be modelled using our previous finite element approach^18^,^19^, we first applied it to a concentric ring structure consisting of four rings designed to reside in a 2D plane with circumferences of 100, 150, 200 and 250 bps, respectively, with inner diameter of 11 nm and outer diameter of 26 nm (Fig. 2a and Supplementary Movie 2). The outer diameter was also measured using atomic force microscopy (AFM) (Supplementary Fig. 11). Although the overall equilibrium 3D structure remained planar, individual helices adopted straightened configurations intervening neighbouring four-way junctions to minimize overall mechanical free energy. In contrast, application of the model to a scaffolded DNA origami structure consisting of nine concentric rings, previously designed and analyzed experimentally using AFM^8^, exhibited significant out-of-plane distortion in addition to in-plane duplex bulging despite the absence of any electrostatic repulsion between neighbouring helices (Fig. 2b and Supplementary Movie 3). Application to a 12-layer hemispherical origami object with outer diameter 44 nm revealed an overall hemispherical structure together with similar local distortions in concentric rings, in addition to a slightly collapsed upper quadrant^8^ (Fig. 2c and Supplementary Movie 4). Our procedure also simulated structural flexibility and thermal fluctuations of these three structures (Supplementary Movies 5–7). These subtle structural features predicted by the model are postulated to have origins in the mismatch in length between intervening DNA duplexes between concentric rings. In particular, our model assumes a 1.85 nm interhelical distance at four-way junctions to be consistent with atomic-level junction models^22^,^23^,^26^, compared with the 2.5 nm assumed previously to design the contour length of each concentric ring^8^ (Supplementary Fig. 8, Supplementary Table 1, and Supplementary Note 4). Differences in the designed radii of two perfectly concentric rings also therefore equals 2.5 nm, leading to contour lengths of 200 and 250 bps of the outermost two layers in the four-layer ring, for example, assuming a 0.34 nm axial rise per bp and an inner ring diameter of 22 nm. This resulting mismatch in contour length consequently leads to the five bulges between two neighbouring immobile four-way junctions in the outermost two layers, as well as the increased curvature in the outermost layer in the four-layer ring. We further assume a ground-state junction angle *J*_twist_=60° corresponding to the free immobile four-way junction, which may further exaggerate this effect. Because the precise pattern of bulging of neighbouring helices and slight collapse observed in the model cannot be resolved experimentally using AFM or transmission electron microscopy, it is impossible at present to determine whether the detailed patterns of predicted local bulging are valid, and if so, whether they are due only to contour mismatch, assumed ground-state junction angle, electrostatic repulsion or some combination of these effects. Notwithstanding, high-resolution 3D structural data from cryo-electron microscopy will be of interest in the future to test these subtle structural predictions for the present, as well as other, programmed DNA assemblies.

### Tile-based assemblies with non-parallel four-way junctions

**Figure 3:**
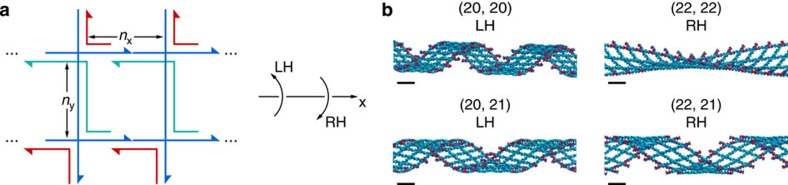
DNA ribbon structures. (**a**) Schematic design of a 2 × 2 lattice with two four-way junctions in both the x- and y-directions. The numbers of bps between neighbouring four-way junctions are marked in the figure as *n*_x_ and *n*_y_ in the x- and y-directions, respectively. The ribbon conformation can be flat (F), left-handed (LH) or right-handed (RH) defined with respect to the x-direction. The 3D structures of the nine 2 × 2 lattices are available in Supplementary Fig. 12. (**b**) Finite element predictions of four 40 × 4 lattices. The centers of the 3D structures of the 40 × 4 lattices are presented. Chirality with respect to the x-direction is denoted at the top of each of the 3D structures. All scale bars are 10 nm.

Next, we tested the ability of our approach to model extended, repeating tile-based structures in which duplexes at four-way junctions are not forced to reside in either parallel or anti-parallel configurations. As a critical evaluation of the empirical four-way junction model, we revisited the tile-based ribbon previously studied by Mao and Seeman^1^,^24^ (Fig. 1). We studied eight variants of the original 21 × 21 bp flat structure by independently varying the lengths of the two duplex arms from 20 to 22 bps. Because each of the four-way junctions in the final assembly are interconnected by B-form DNA duplexes that resist bending, twisting and stretching, over- and under-winding and stretching induced by these mismatches in tile dimensions are expected to result in ribbon structures that deviate from the flat, ground-state structure observed for the 21 × 21 bp case^24^. Geometric arguments based on the natural pitch of B-form DNA suggest that a single deletion (insertion) in the x-direction, *n*_x_=20 (22) bp, will result in a left (right)-handed twist about the x-direction (Fig. 3a and Supplementary Fig. 12). In contrast, a single deletion (insertion) in the y-direction, *n*_y_=20 (22) bp, will result in a right (left)-handed twist about the x-direction (Fig. 3a and Supplementary Fig. 12). Following this geometric argument, six tile designs consist of non-conflicting handedness or chirality, whereas two cases, (*n*_*x*_, *n*_*y*_)=(20, 20) and (22, 22) bp, consist of conflicting handedness (Fig. 3a and Supplementary Fig. 12). Interestingly, the model predicts that the (20, 20) design is left-handed about the x-direction, whereas the (22, 22) design is right-handed. In contrast, the model predicts a handedness in each of the other six cases that is consistent with the preceding geometric argument (Supplementary Fig. 12). Experimentally, we synthesized N × 4 lattices instead of N × 2 lattices to resolve their handedness within the limited resolution of AFM. Synthesized structures agreed with model predictions for the (21, 21), (22, 21) and (22, 22) designs in which folding was successful, whereas low synthesis yields of the (20, 20) and (20, 21) designs in diverse buffer conditions precluded a determination of ribbon structure (Supplementary Figs 13 and 14). Although resolving the origin of this failure to self-assemble remains for future work, an intriguing possibility is that the (20, 20) and (20, 21) designs with a left-handed twist about the x-direction force the immobile four-way junctions, which are flanked by four bps T-A, C-G, A-T and G-C and first introduced by Seeman^1^, into energetically unstable conformations.

### 3D crystalline assemblies

**Figure 4:**
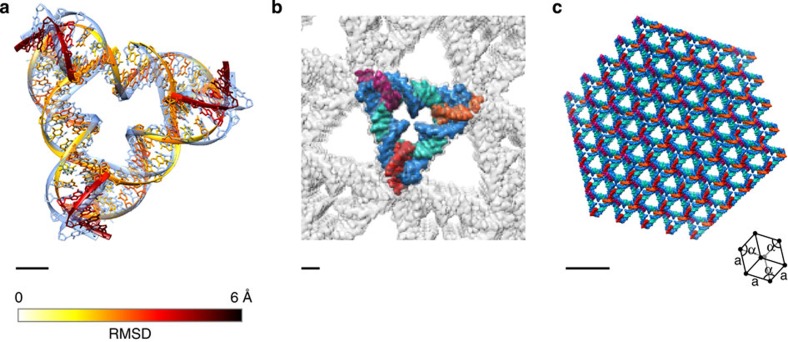
3D crystal lattice of a tensegrity motif. (**a**) Zoomed-in view of the central crystal cell aligned with the crystallographic structure PDB ID: 3GBI (light blue). Each nucleotide in the crystal structure predicted by the model is coloured according to its RMSD on a per-nucleotide basis with respect to the corresponding nucleotide in the crystallographic structure. (**b**) The zoomed-in central unit cell coloured according to DNA strands with the surrounding unit cells shown in semi-transparent rendering. (**c**) The overall crystal structure of PDB ID 3GBI^14^ predicted using the finite element model applied to a 5 × 5 × 5 cell. The lower right inset shows the size and orientation of a unit cell, where crystal axes and angles are denoted by a and α, respectively. Three orthogonal views of each panel are depicted in Supplementary Fig. 15. Scale bars are 1 nm (**a**,**b**) and 10 nm (**c**).

Finally, we tested the ability of our modelling approach to be applied to extended 3D DNA-based crystals in which the immobile four-way junction was previously designed in a tensegrity motif to form a rhombohedral crystal^14^. We applied our junction-based model to predict the 3D structure of the crystal assuming the same ground-state geometry and mechanical properties of the junction as assumed above (Fig. 4, Supplementary Fig. 15, and Supplementary Movie 8). Comparison of the predicted central cell of a 5 × 5 × 5 cell structure with the published structural coordinates from PDB ID 3GBI reveals an average root mean square deviation (RMSD) of 3.2 Å over all atoms, with a maximum of 5.3 Å and minimum of 1.9 Å on a per-nucleotide basis (Fig. 4a and Supplementary Fig. 15). Approximately 80% of the nucleotides have an RMSD that is lower than the overall crystal structure resolution of 4.0 Å. Interestingly, altering the ground-state angle *J*_twist_ and three rotational stiffnesses assumed for the four-way junction model does not significantly affect the predicted equilibrium, ground-state structure (Supplementary Figs 16–19). Note that the concentric ring structures and the tile-based structures are generally more sensitive to the rotational stiffnesses (Supplementary Figs 20–27). Although on the one hand this indicates that our finite-element-based structural prediction is similar to a considerably simpler, purely geometric model, it also suggests that the designed crystalline motif is highly robust to variations in empirically assumed ground-state junction angles and mechanical properties. This may offer an explanation for why this motif crystallized successfully in the first place. In either case, our computational framework lays the foundation for sequence-based 3D modelling of DNA-based crystalline materials consisting of one or more types of multi-way junctions.

## Discussion

We present a computational framework to predict the 3D structure of complex, high molecular weight nucleic-acid assemblies programmed using immobile multi-way junctions. Modelling immobile junctions and interconnecting DNA duplexes as mechanical elements with empirically known properties renders the model applicable to arbitrary sequence topologies used to design nucleic-acid assemblies. Application to a diversity of constructs designed using the canonical four-way junction affirms its ability to predict 2D- and 3D-curved objects containing duplexes that are topologically closed as well as locally nonparallel, which is impossible using our previously developed framework^18^,^19^.

Important limitations of our model include the fact that ground-state geometries and flexibilities of multi-way junctions must be known empirically, and dynamic inter-conversions between distinct isomeric or other competing conformational states are ignored together with electrostatic interactions. Additional assumptions include constant geometric and mechanical properties of DNA duplexes, which in reality depend on their local sequence composition, temperature and solvent conditions, as well as degree of external forcing. Notwithstanding, because the present methodology is based on a coarse-grained mechanical model implemented using the finite element method, it is capable of predicting the 3D global architecture of high molecular weight nucleic acid assemblies in a computationally efficient manner, within the limits imposed by the preceding assumptions.

Integration of this procedure into our online structure-prediction framework CanDo will provide a broadly accessible 3D structure-prediction framework that can be refined and extended in the future using high-resolution structural feedback, which can include the detailed exploration of junction geometries, flexibilities and stabilities, together with their dependence on sequence, solvent and structural context. For example, in the design of parallel versus anti-parallel DNA duplexes constrained by densely organized crossovers, it is likely that four-way junctions adopt different ground-state angles than the free, 60° right-handed *J*_twist_ assumed here^25^,^26^. Exploring the complex dependence of junction properties on these factors may be the subject of future work. Because DNA offers the ability to program 3D nanoscale materials of high structural fidelity at the nanometer-scale, refining the present modelling framework for application to these structural contexts will be important to enable the rational design of functional nanoscale materials for diverse applications in biomolecular and materials science^21^,^36^,^37^.

## Methods

### Sequence and secondary structure definition and parsing

Programmed secondary structure and sequence topology of DNA assemblies are extracted from a design file provided in Tiamat format^28^. The routing of individual DNA strands is read as a directed graph in which each vertex is a nucleotide, and edges represent phosphate-sugar backbones and Watson–Crick basepairings. Specifically, for any given nucleotide in the assembly, this graph uniquely identifies the nucleotide in the 5′-direction, that in the 3′-direction and the complementary nucleotide, if existing. The identity of each nucleotide is obtained from the sequence information stored in the Tiamat file (Supplementary Note 1).

Four-way junctions and their connectivities via DNA duplexes are identified from the preceding directed graph. We developed an algorithm to traverse the directed graph and identify all branch points, that is, the phosphate-sugar backbones flanked by two arms of a four-way junction. The same algorithm then finds all pairs of branch points connected by DNA duplexes, the numbers of bps between each pair, and positions of nicks in DNA duplexes (Supplementary Note 1). Note that the above approach can also be applied to read sequence and topology from design files in caDNAno format^20^, which places duplexes in parallel along canonical honeycomb or square lattices in contrast to the Tiamat format that allows duplexes to be placed in any position and orientation in 3D space.

### 3D structure calculation

The finite element model is defined from the sequence-based programmed DNA topology, where DNA assemblies are modelled as a set of interconnected ‘stacked-X’ immobile four-way junctions connected by B-form DNA duplexes. B-form duplexes are modelled as isotropic linear elastic beams, where each bp is modelled as a finite element node with six degrees of freedom (DOFs), and a two-node Hermitian beam element is generated between two stacking bps with the initial axial length 0.34 nm and the initial twist angle of 360°/10.5=34.29°. The stretch modulus 1,100 pN, bend modulus 230 pN nm^2^ and twist modulus 460 pN nm^2^ of the beam are set to their experimental values for B-form DNA, as previously performed^18^,^19^. Nicks in dsDNA are modelled by the same beam elements with either identical or reduced bend and twist moduli^19^ (Supplementary Note 2).

B-form DNA bps flanking immobile four-way junctions are chosen to be separated by 1.85 nm^25^ and have a junction angle of 60° in a right-handed sense^24^,^31^,^32^. Each DNA helix has three translational and rotational DOFs relative to its neighbour at the center of the four-way junction to model structural flexibility of the junction. A reference frame for the four-way junction is placed at its center with axes **e**_1_, **e**_2_ and **e**_3_ (Supplementary Figs 7 and 8 and Supplementary Note 4). The principal flexible DOF is modelled to be the scissor-like interhelical angle *J*_twist_ about **e**_3_. The harmonic stiffness is 1,353 pN nm rad^−1^ about **e**_1_, 1,353 pN nm rad^−1^ about **e**_2_ and 135.3 pN nm rad^−1^ about **e**_3_ (Supplementary Table 1 and Supplementary Note 4). One of the two possible isomeric states, defined by neighbouring stacking interactions of the duplex arms (Supplementary Fig. 6), is assigned for each four-way junction by first calculating angles between the four arms from the initial Cartesian coordinates of the nucleotides in the design file and then determining the neighbouring stacking interactions. A duplex arm is considered to be coaxially stacked with a neighbouring arm if the angle between the former and the latter is closer to 180° than the angle between the former and the other neighbouring arm. Once assigned, the isomeric state of a junction is immutable in the finite element equilibrium ground-state calculation.

The 3D mechanical ground-state solution structure is computed iteratively using the geometrically nonlinear finite element solution procedure available in the commercial finite element software ADINA (ADINA R&D, Watertown, MA, USA). The simulation begins by simultaneously applying forces and moments to each pair of ends of four-way junctions that are connected by dsDNA to obtain geometric compatibility in which the two arm ends coincide in their position and orientation to form a continuous duplex of B-form DNA (Supplementary Figs 5, 28–30 and Supplementary Note 3). During this process, the full finite element model is simultaneously relaxed to adopt its mechanical equilibrium structure. Normal mode analysis for the equilibrium 3D structure yields structural flexibility and thermal fluctuations at a given finite temperature as root mean square fluctuations in all DOFs of all bps^19^,^34^.

### Parameter sensitivity analysis

A sensitivity analysis was performed to evaluate the effects of the rotational junction stiffness parameter *k*_twist_ and the stiffness of DNA nicks on the computed 3D structures. 3D structures were first calculated using *k*_twist_ equal to the standard value multiplied by a factor *a* with the bend and twist moduli of nicked DNA equal to the standard values multiplied by a factor *b* (Supplementary Figs 20–27). The 3D structures of the concentric rings (Supplementary Figs. 20–22) are largely insensitive to the rotational junction stiffness *k*_twist_ and the bend and twist moduli of nicks. In contrast, the (22, 21), (20, 21), (22, 22) and (20, 20) lattices exhibit relatively high sensitivity to the factors *a* and *b* (Supplementary Figs 24–27). Notably, the chirality of the (22, 22) lattice is also sensitive to the effective stiffness of nicks (Supplementary Fig. 26). RMSDs with the corresponding reference atomic models are presented in Supplementary Table 2.

### Atomic structure generation

Atomic coordinates of DNA are generated using the translational and rotational coordinates of the finite element model. The atomic model is generated by placing standard reference atomic structures for four Watson–Crick bps at node positions and orientations^34^. According to the crystallographic structure of a free four-way junction (PDB ID: 1DCW)^23^, the computational framework then adjusts the coordinates of the phosphate and sugar at the crossover site in the backbone of a crossing strand in a four-way junction by rotating them by 45° about the vector from the C1′ atom to the N1 atom in the same nucleotide.

### Preparation of assembled nanostructures

All DNA strands were purchased from Integrated DNA Technologies Inc. ( www.IDTDNA.com) at a 25 or 100 nmole synthesis scale. All strands were further purified using denaturing PAGE gel. The sequences of DNA oligos used to form all the structures were designed using the program Tiamat^28^. A one-step annealing reaction was used to form each structure. The strands for four-way junctions in each design are mixed with final concentration as 0.2 mM in 1xTAE-Mg^2+^ buffer (20 mM Tris, pH 7.6, 2 mM EDTA, 12.5 mM MgCl_2_). The oligonucleotide mixture was annealed in a thermocycler that was programmed to cool from 95 °C to 4 °C in total 12 h: 94 °C to 86 °C at 4 °C per 5 min; 85 °C to 70 °C at 1 °C per 5 min; 70 °C to 40 °C at 1 °C per 15 min; 40 °C to 25 °C at 1 °C per 10 min; and then held at 4 °C.

### AFM imaging

1 ml sample was deposited onto a slide of freshly pealed mica (Ted Pella, Inc.) and left for 0.5 min for adsorption. 80 ml 1x TAE-Mg^2+^ buffer was added on top of the sample and an extra 40 ml of the same buffer was added on the window of the AFM tip. The sample was scanned in the scan-analysis in fluid mode using AFM (Dimension FastScan, Bruker Corporation) with SCANASYST-FLUID+ tips (Bruker, Inc.).

## Author contributions

K.P., D-N.K. and M.B. conceived of the finite element modelling and structure-prediction approach. M.R.A. performed MD simulations and analyses. K.P. implemented the primary and secondary structure parsing algorithm. K.P. and D-N.K. implemented the finite element model and structure-prediction procedure. K.P. applied the model to compute the modelling results and processed the results to make the figures. F.Z. and H.Y. conceived of the experimental assay. F.Z. implemented the experimental assay, generated the experimental data, and processed the results to make the experimental figures. All authors discussed the results and their presentation. K.P., D-N.K. and M.B. wrote the manuscript. All authors commented on and edited the manuscript.

## Additional information

**How to cite this article:** Pan, K. *et al.* Lattice-free prediction of three-dimensional structure of programmed DNA assemblies. *Nat. Commun.* 5:5578 doi: 10.1038/ncomms6578 (2014).

## Supplementary Material

Supplementary Figures, Tables, Methods and References Supplementary Figures 1-30, Supplementary Tables 1-2, Supplementary Notes 1-4 and Supplementary References.

Supplementary Movie 1 Movie showing the structure prediction process that is based on the initial finite element model configuration followed by restorative forces and moments applied to the ends of topologically connected duplexes to force their alignment into the final equilibrium solution shape. The final frame of the movie is the same as the final configuration shown in Figure 2a.

Supplementary Movie 2 Movie showing the predicted three-dimensional atomic structure of the 4-layer ring from Figure 2a. The atomic structure is first rotated by ±90° about the horizontal axis and then ±90° about the vertical axis.

Supplementary Movie 3 Movie showing the predicted three-dimensional atomic structure of the 9-layer ring origami from Figure 2b. The structure is first rotated by ±90° about the horizontal axis and then ±90° about the vertical axis.

Supplementary Movie 4 Movie showing the three-dimensional solution structure of the 12-layer hemispherical origami from Figure 2c. The structure is first rotated by 360° about the horizontal axis and then by 360° about the vertical axis.

Supplementary Movie 5 Movie showing the thermal fluctuations (at 298 K) of the 4-layer ring from Figure 2a in three orthogonal views.

Supplementary Movie 6 Movie showing the thermal fluctuations (at 298 K) of the 9-layer ring origami from Figure 2b in three orthogonal views.

Supplementary Movie 7 Movie showing the thermal fluctuations (at 298 K) of the 12-layer hemispherical origami from Figure 2c in three orthogonal views.

Supplementary Movie 8 Movie showing the predicted three-dimensional solution structure of the tensegrity crystalline lattice from Figure 4. The overall lattice is first rotated by ±90° about the horizontal axis and then ±90° about the vertical axis, followed by zoom-in to the central unit and the same series of rotations.

Supplementary Dataset 1 Tiamat and PDB files for 3D crystal lattice.

Supplementary Dataset 2 Tiamat and PDB files for 4-layer ring.

Supplementary Dataset 3 Tiamat and PDB files for 9-layer ring origami.

Supplementary Dataset 4 Tiamat and PDB files for 12-layer hemispherical origami.

Supplementary Dataset 5 Tiamat and PDB files for 40×2 ribbon with [n_x_, n_y_] = [21, 21] bps.

Supplementary Dataset 6 Tiamat and PDB files for 40×4 ribbon with [n_x_, n_y_] = [21, 21] bps.

Supplementary Dataset 7 Tiamat and PDB files for 40×2 ribbon with [n_x_, n_y_] = [22, 21] bps.

Supplementary Dataset 8 Tiamat and PDB files for 40×4 ribbon with [n_x_, n_y_] = [22, 21] bps.

Supplementary Dataset 9 Tiamat and PDB files for 40×2 ribbon with [n_x_, n_y_] = [20, 21] bps.

Supplementary Dataset 10 Tiamat and PDB files for 40×4 ribbon with [n_x_, n_y_] = [20, 21] bps.

Supplementary Dataset 11 Tiamat and PDB files for 40×2 ribbon with [n_x_, n_y_] = [22, 22] bps.

Supplementary Dataset 12 Tiamat and PDB files for 40×4 ribbon with [n_x_, n_y_] = [22, 22] bps.

Supplementary Dataset 13 Tiamat and PDB files for 40×2 ribbon with [n_x_, n_y_] = [20, 20] bps.

Supplementary Dataset 14 Tiamat and PDB files for 40×4 ribbon with [n_x_, n_y_] = [20, 20] bps.
