# Supplementary material for: Lattice-free prediction of three-dimensional structure of programmed DNA assemblies
Source: Nat Commun. 2014 Dec 3;5:5578. doi: 10.1038/ncomms6578 (PMC4268701; doi:10.1038/ncomms6578)
Supplement: Supplementary Figures, Tables, Methods and References — Supplementary Figures 1-30, Supplementary Tables 1-2, Supplementary Notes 1-4 and Supplementary References. [file ncomms6578-s1.pdf]

Supplementary Figures

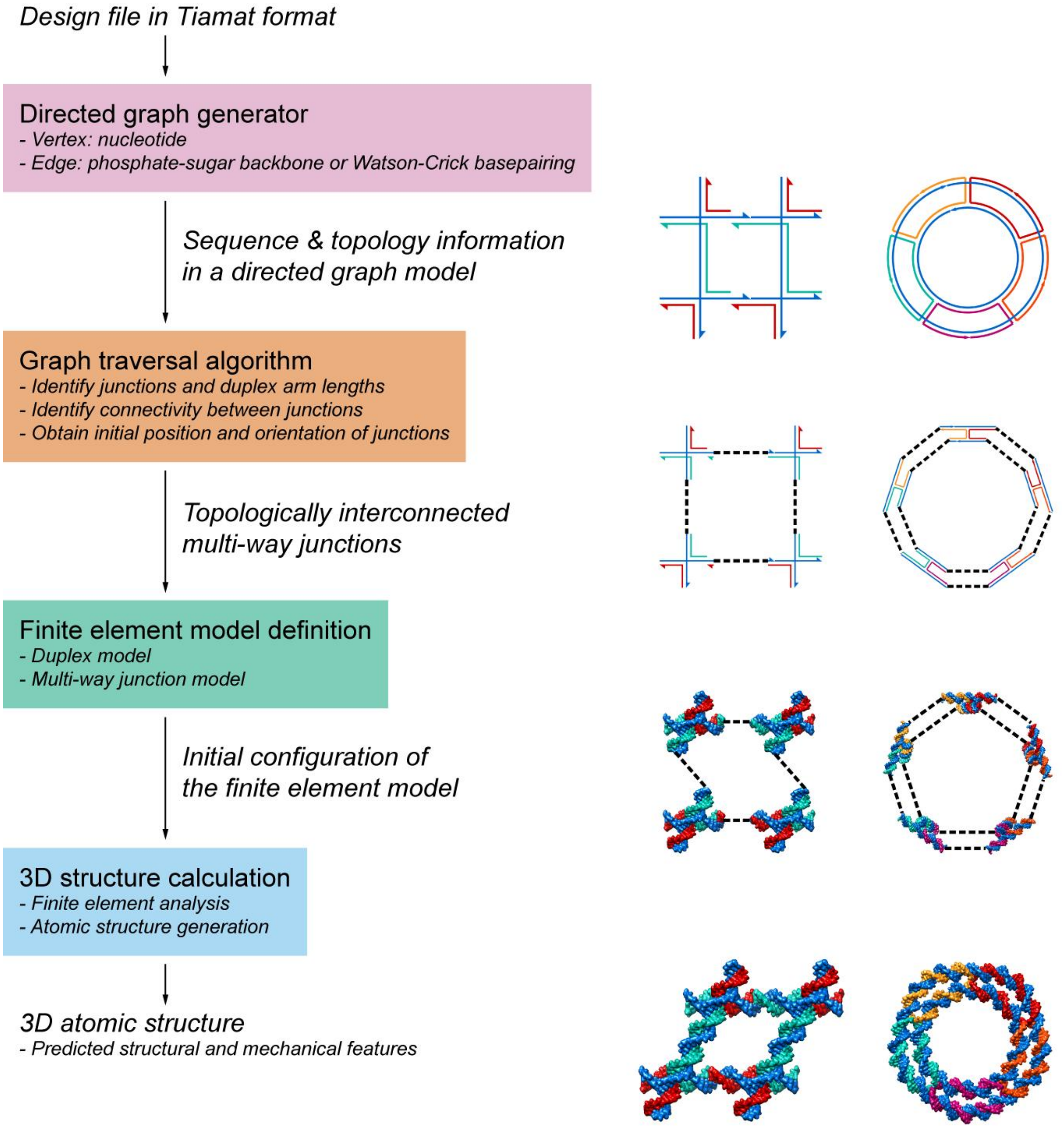

Supplementary Figure 1 | Computational framework to parse DNA sequence and topology and predict 3D structure of programmed DNA assemblies. The flowchart on the left depicts the workflow in this framework, which is demonstrated with two exemplary designs on the right, including a 2×2 four-way junction square lattice and a two-layer ring. The ends of topologically connected duplexes are connected by dashed lines.

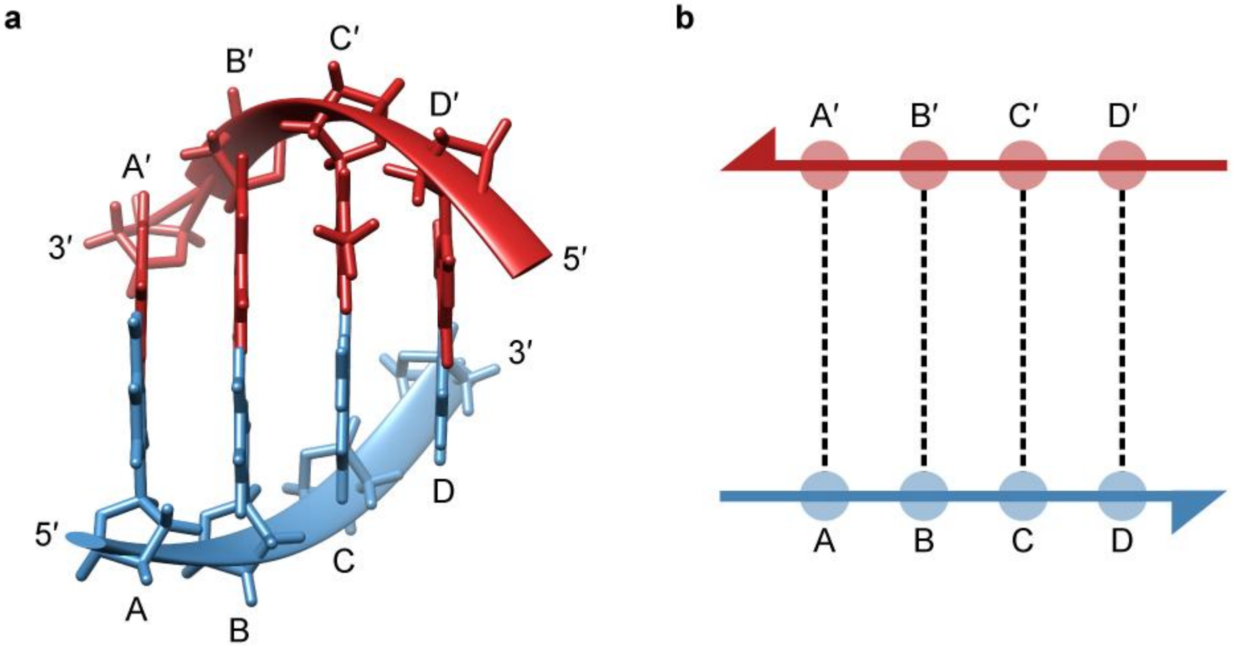

Supplementary Figure 2 | Graph representation of DNA. (**a**) A B-form DNA duplex consisting of eight paired nucleotides. (**b**) Equivalent graph representation of the same DNA duplex with nucleotides represented as nodes. Dashed lines indicate Watson-Crick basepairing and solid lines indicate phosphate-sugar backbone.

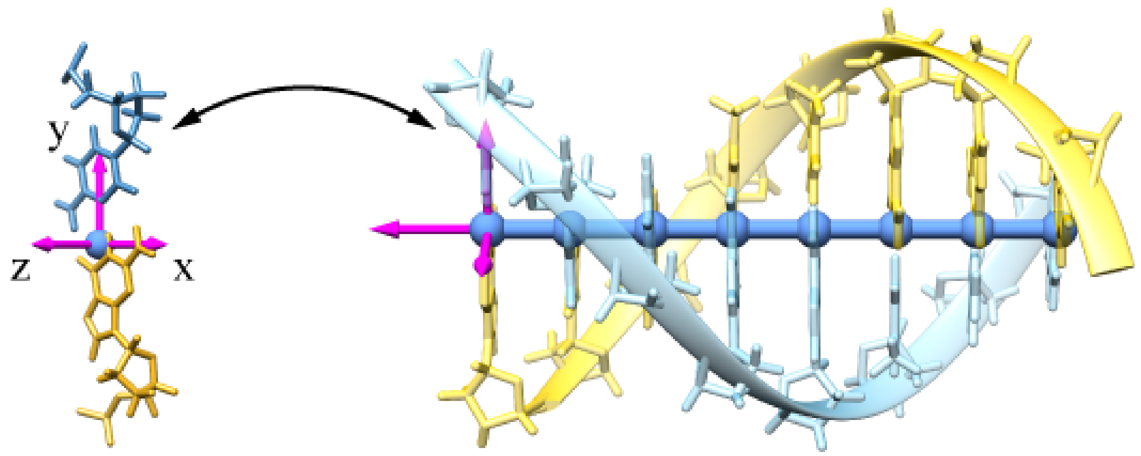

Supplementary Figure 3 | Finite element model of B-form DNA. Standard reference atomic coordinates of a bp (colored in blue and yellow) determine the position (blue sphere) and orientation (magenta axes) of the corresponding finite element node. Finite element nodes are connected by beam elements (blue cylinders) to form the duplex shown at right.

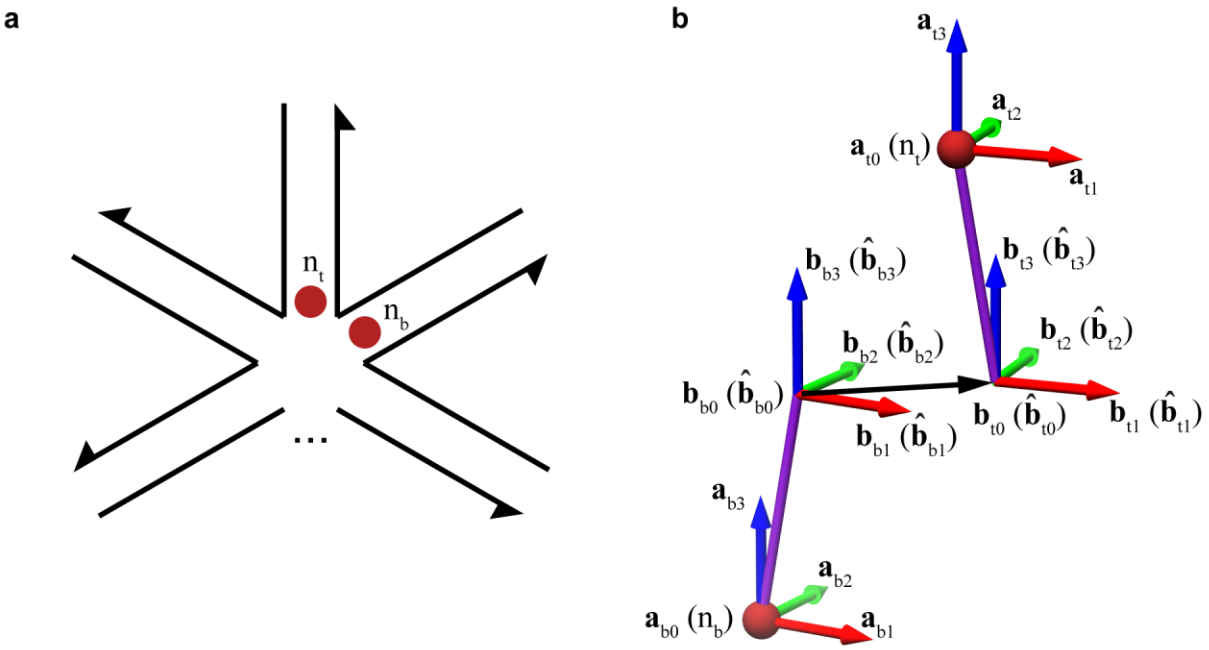

Supplementary Figure 4 | Alignment element used to implement generic junction models. (**a**) Generic multi-way junctions consist of N duplexes topologically interconnected via phosphate-sugar backbones. (**b**) The finite element model consists of alignment elements connecting two nodes denoted n_b and n_t (red spheres). Each node is associated with A- and B-triads. The A-triad of node n_b has the center $\mathbf{a}_{b0}$ at n_b and three axes $\mathbf{a}_{b1}$, $\mathbf{a}_{b2}$, and $\mathbf{a}_{b3}$. Purple cylinders represent rigid connection between A- and B-triads of the same node. The B-triad of n_b has the center $\mathbf{b}_{b0}$ ($\hat{\mathbf{b}}_{b0}$) and three axes $\mathbf{b}_{b1}$ ($\hat{\mathbf{b}}_{b1}$), $\mathbf{b}_{b2}$ ($\hat{\mathbf{b}}_{b2}$), and $\mathbf{b}_{b3}$ ($\hat{\mathbf{b}}_{b3}$), where the vectors without parentheses are defined with respect to the global reference frame, and those in parentheses are to the A-triad of the same node. The A- and B-triads of node n_t are defined similarly. To illustrate, the black arrow indicates the translational misalignment between n_b and n_t.

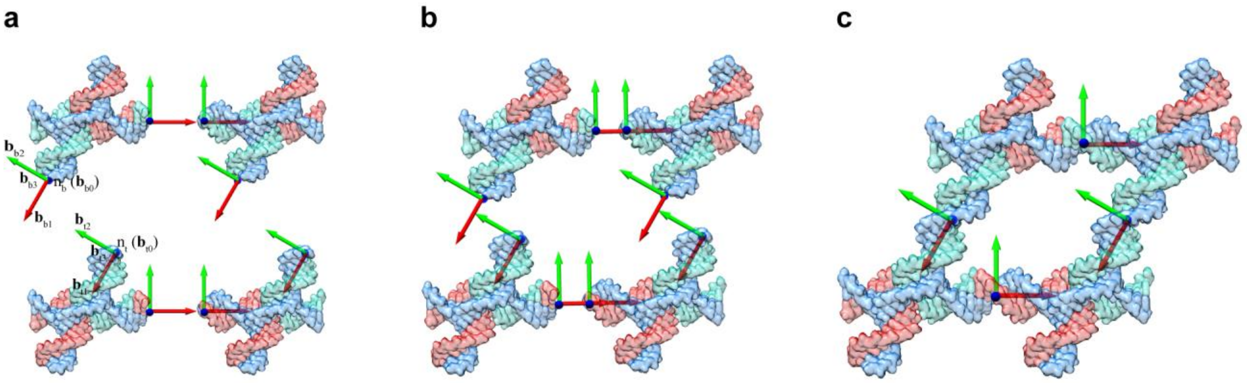

Supplementary Figure 5 | Connections between termini of four-way junction arms in the 2×2 four-way junction square lattice shown in Fig. 1. Triads are shown in the (**a**) initial configuration, (**b**) intermediate configuration, and (**c**) final configuration. The three axes of each triad are colored in red, green, and blue (perpendicular to the figure plane), respectively. In the initial configuration, the centers and axes are marked in one pair of triads rigidly attached to the two nodes to be aligned.

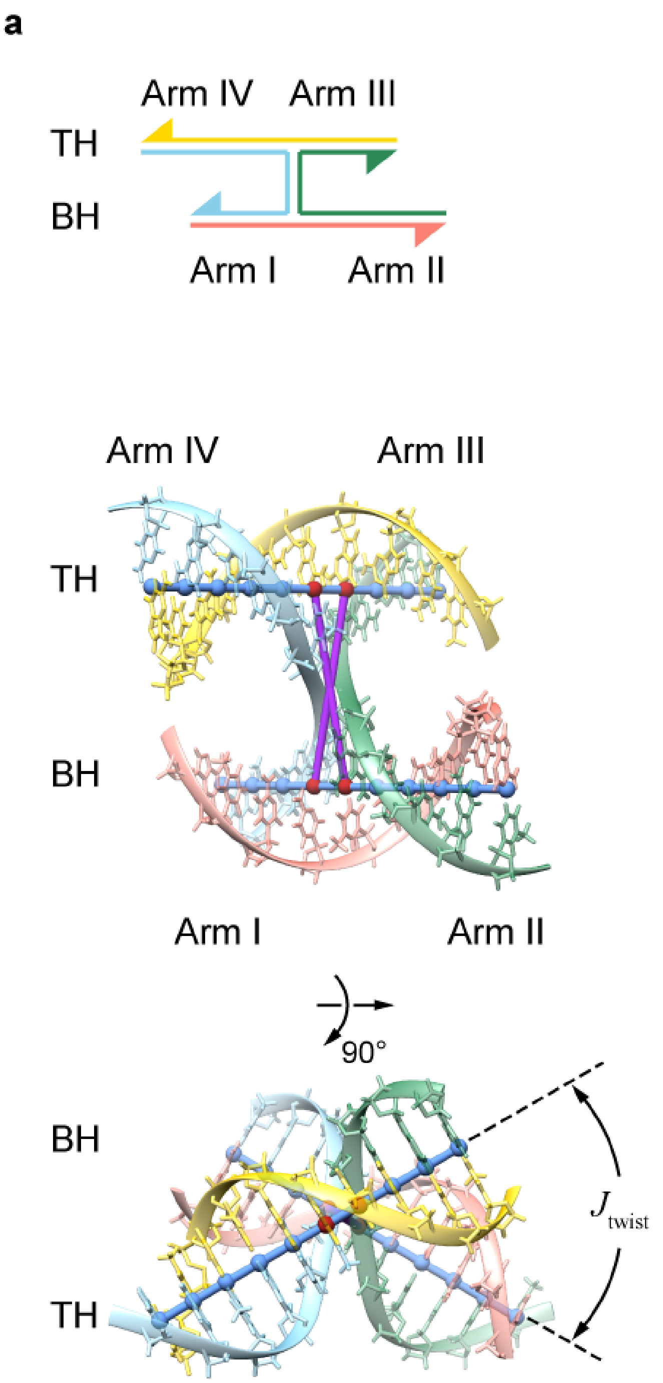

Supplementary Figure 6 | Overview of the "stacked-X" immobile four-way junction model. (**a**) (Upper) Sequence and topology information of a "stacked-X" four-way junction consisting of two duplexes of 10 bps length each, namely the bottom helix (BH) and top helix (TH). (Lower) The finite element model in two orthogonal views of the same junction is generated from the sequence and topology information and comprises two beams for two stacked duplexes, i.e. BH and TH. Two finite element nodes (red spheres) corresponding to two bps connected by a crossing strand are connected by an alignment finite element (purple). (**b**) The same four-way junction in the alternative isomeric state.

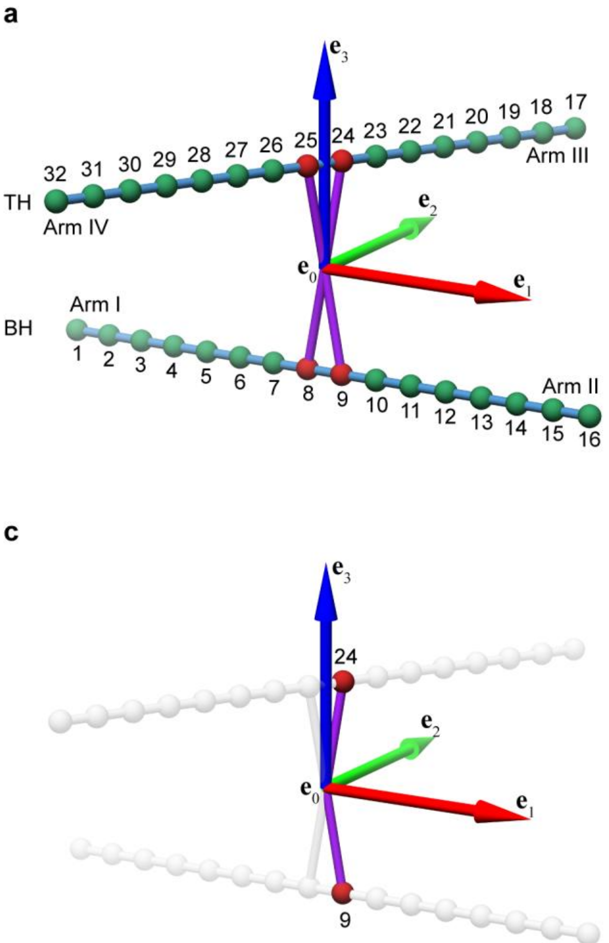

Supplementary Figure 7 | Finite element model for a 32-bp "stacked-X" four-way junction in its ground state. (**a**) The BH and TH in the four-way junction are modeled as two beams defined using a reference frame with its center position $\mathbf{e}_0$ and three directional vectors $\mathbf{e}_1$, $\mathbf{e}_2$, and $\mathbf{e}_3$. Spheres represent finite element nodes describing the positions and orientations of bps, light blue lines represent beam elements, and purple lines represent alignment elements. The four nodes at the crossover site are colored in red. (**b** and **c**) Two alignment elements are highlighted separately. Two triads centered at $\mathbf{b}_{b0}$ and $\mathbf{b}_{t0}$ are rigidly connected to the two red nodes in the BH and TH, respectively, and coincide with the reference triad at the ground state. (**d**) Calculation of misalignments from the two triads rigidly connected to the nodes in the BH and TH. All the nodes and beam elements are omitted for clarity.

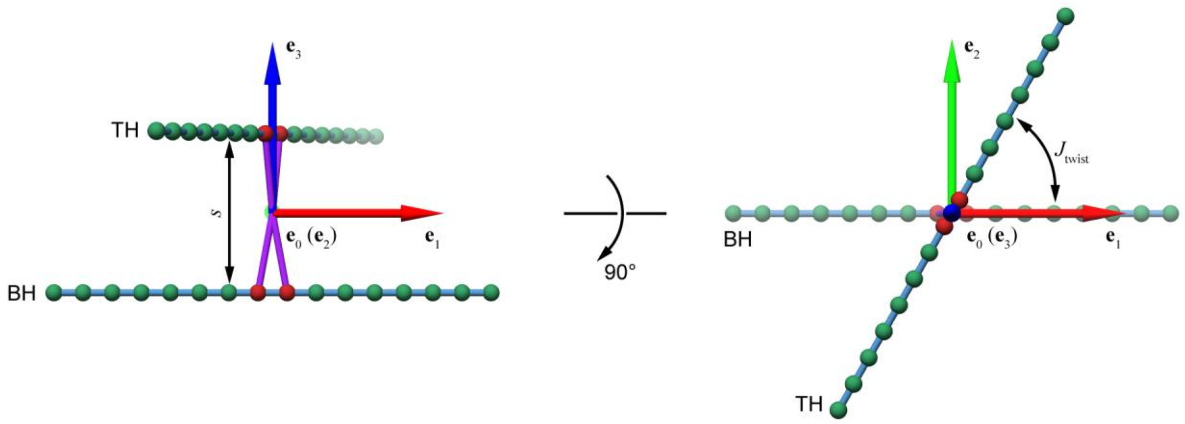

Supplementary Figure 8 | Geometric parameters of the finite element four-way junction model. This figure defines the interhelical distance s and the interhelical angle J_{twist} between the BH and TH in two orthogonal views, with regard to the reference frame defined by its center position $\mathbf{e}_0$ and three directional vectors $\mathbf{e}_1$, $\mathbf{e}_2$, and $\mathbf{e}_3$.

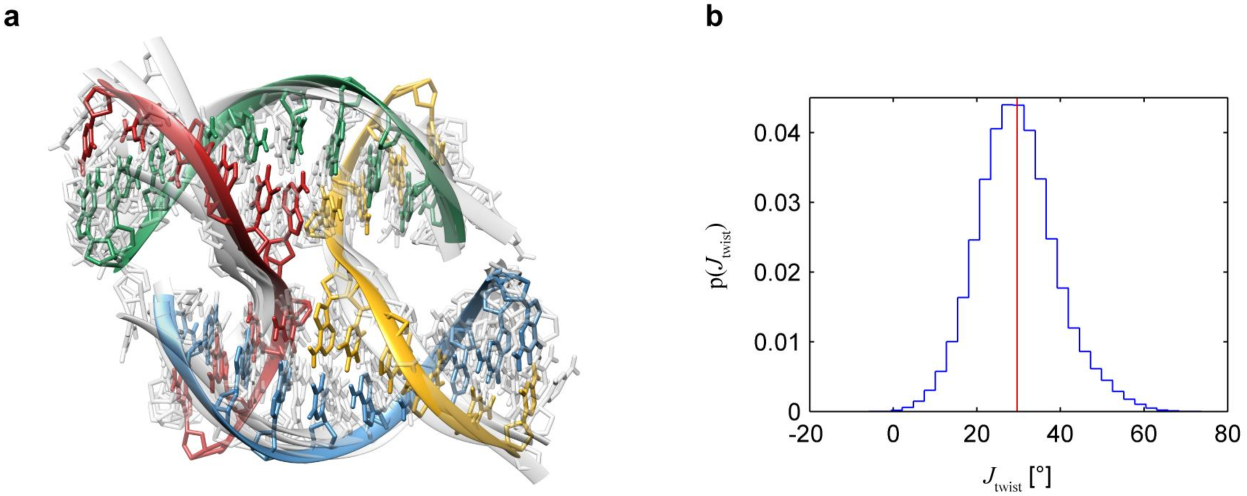

Supplementary Figure 9 | Estimating the junction stiffness coefficient k_{twist} using all-atom MD simulations. (**a**) The crystallographic structure of 1DCW aligned with four snapshots from MD trajectories shown in semi-transparent rendering. (**b**) Probability densities of J_{twist}. The red vertical line denotes the mean of the distribution.

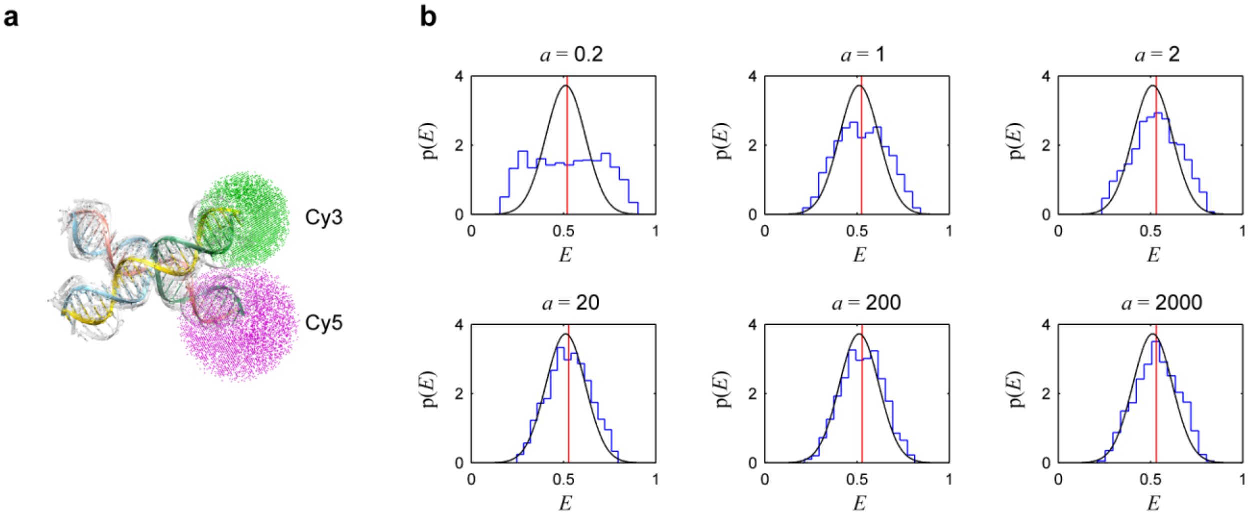

Supplementary Figure 10 | Validation of k_{twist} using FRET efficiency data. (**a**) The flexible four-way junction in the specific isomeric state to which the Cy3 (donor) and Cy5 (acceptor) dyes covalently conjugated. The AVs of Cy3 and Cy5 dyes are represented by 3D point clouds. The ground-state junction configuration is aligned with four snapshots from the finite element normal mode analysis shown in semi-transparent rendering. (**b**) Distributions of FRET efficiencies between the dyes in six simulations in which the standard value of rotational junction stiffness k_{twist} is multiplied by a = 0.2, 1, 2, 20, 200, and 2000, respectively. The black curves are the probability density of FRET efficiencies measured in experiment and plotted as a Gaussian with mean μ_E = 0.51 and standard deviation σ_E = 0.11, as reported in the literature[1]. The simulated probability densities and their means are colored in blue and red, respectively.

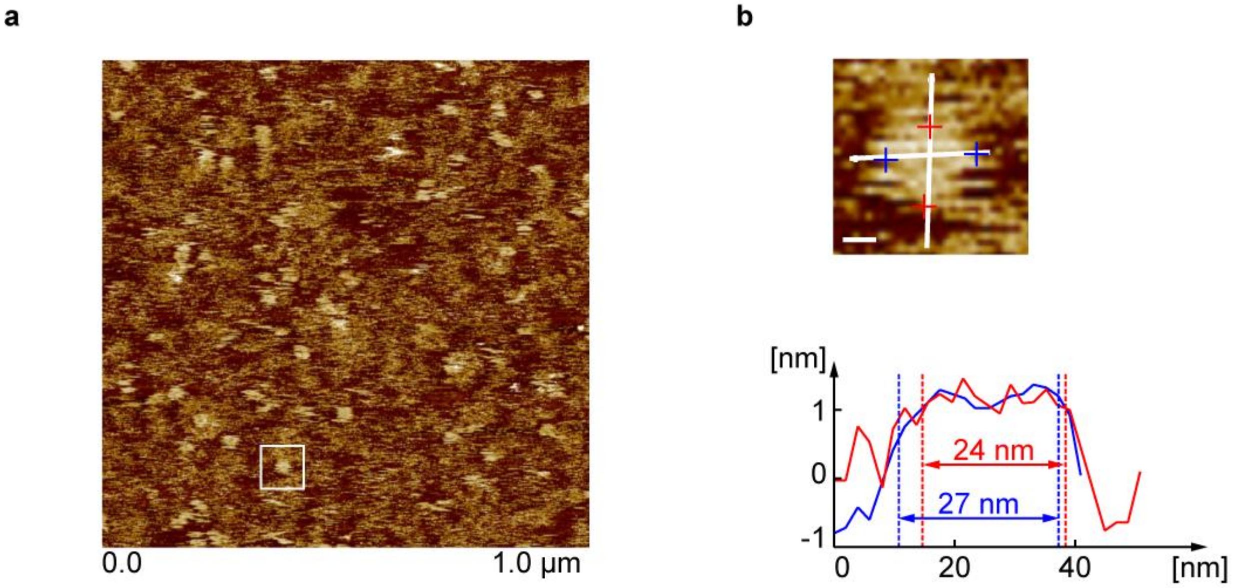

Supplementary Figure 11 | AFM image of the 4-layer ring. (**a**) AFM image of the 4-layer ring with 1.0 μm along each side. The nanostructure in the white box is zoomed in in the next panel. (**b**) The diameter of the 4-layer ring is measured using the AFM image of this single nanostructure. The scale bar is 10 nm. The measured AFM height data along the horizontal and vertical white lines in the AFM image are plotted in blue and red, respectively. The section with 2 nm thickness is selected as the target structure which is between the blue and red crosses in the AFM image, corresponding to the vertical dashed lines in the height data.

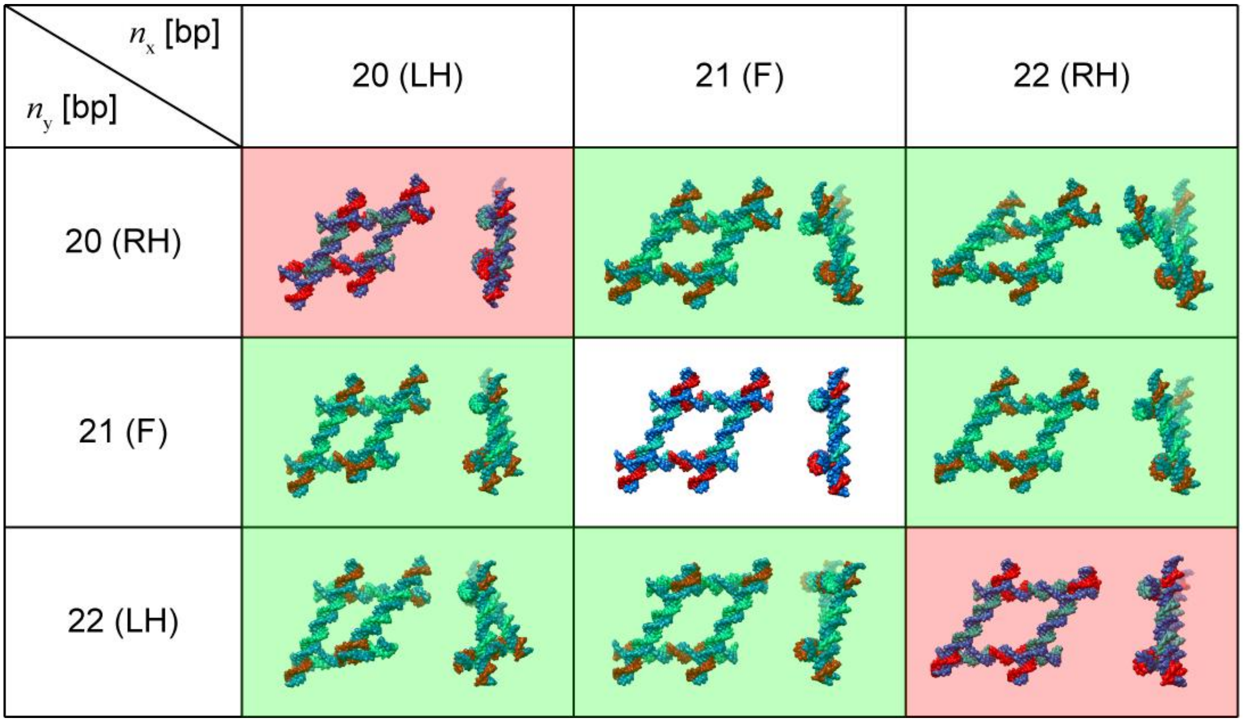

Supplementary Figure 12 | 3D structures in two orthogonal views of nine designs of the 2×2 tile lattice. The schematic design is in Fig. 3a. The effect of a single deletion or insertion on the chirality with respect to the x-direction defined in Fig. 3 is given in parentheses. Designs of non-conflicting and conflicting x- and y-arm induced chirality are colored in green and red, respectively. The original (21, 21) flat tile is un-colored.

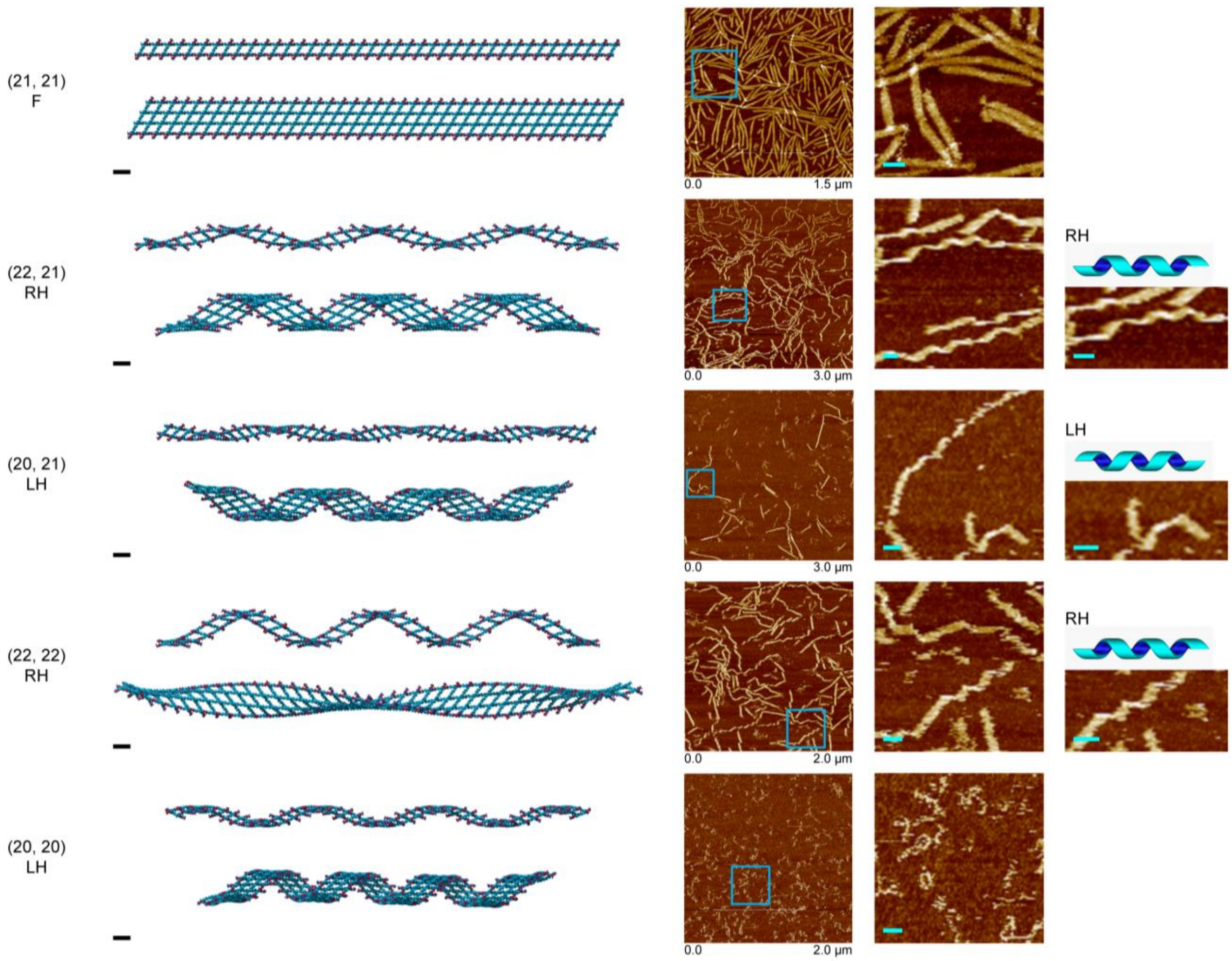

Supplementary Figure 13 | Finite element 3D structures of 40×2 and 40×4 lattices. Junctions are separated in the x- and y-direction by (n_x, n_y) = (21, 21), (22, 21), (20, 21), (22, 22), and (20, 20) bps. Chirality with respect to the x-direction is marked to the left of the 3D structures. Also shown are three columns of AFM images of the *N*×4 lattices with varying lengths in the x-direction. In the first column, sizes of AFM images along each side are given under the images. Images inside the blue boxes are zoomed in, creating the AFM images in the second column. In the third column, AFM images of three designs are compared to geometric models of right-handed (RH) and left-handed (LH) helices, showing chirality of the 3D structures. Scale bars are 10 nm (atomic models) and 50 nm (AFM images).

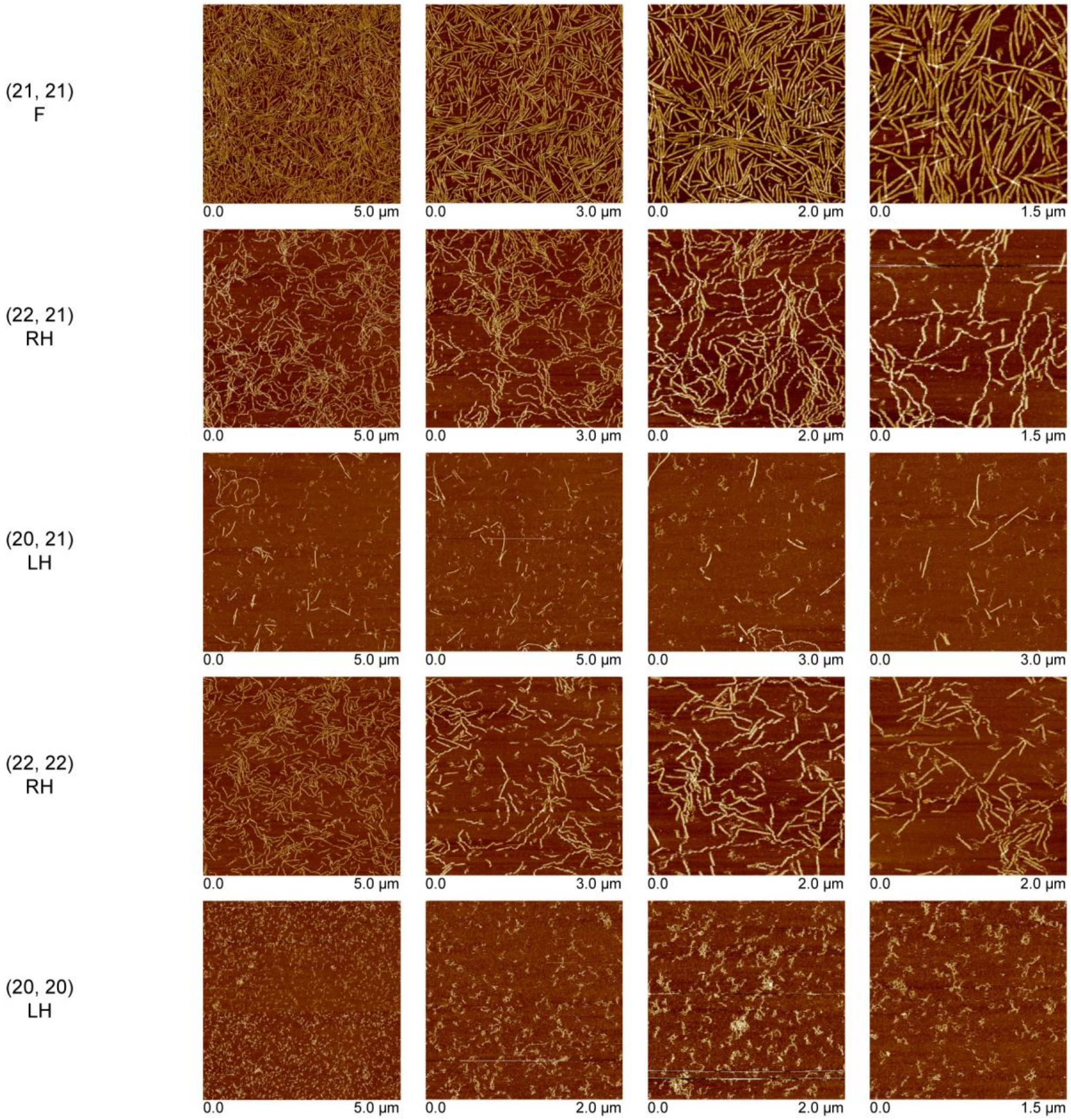

Supplementary Figure 14 | AFM images of the *N*×4 lattices with varying lengths in the x-direction. Sizes of AFM images along each side are given under the images.

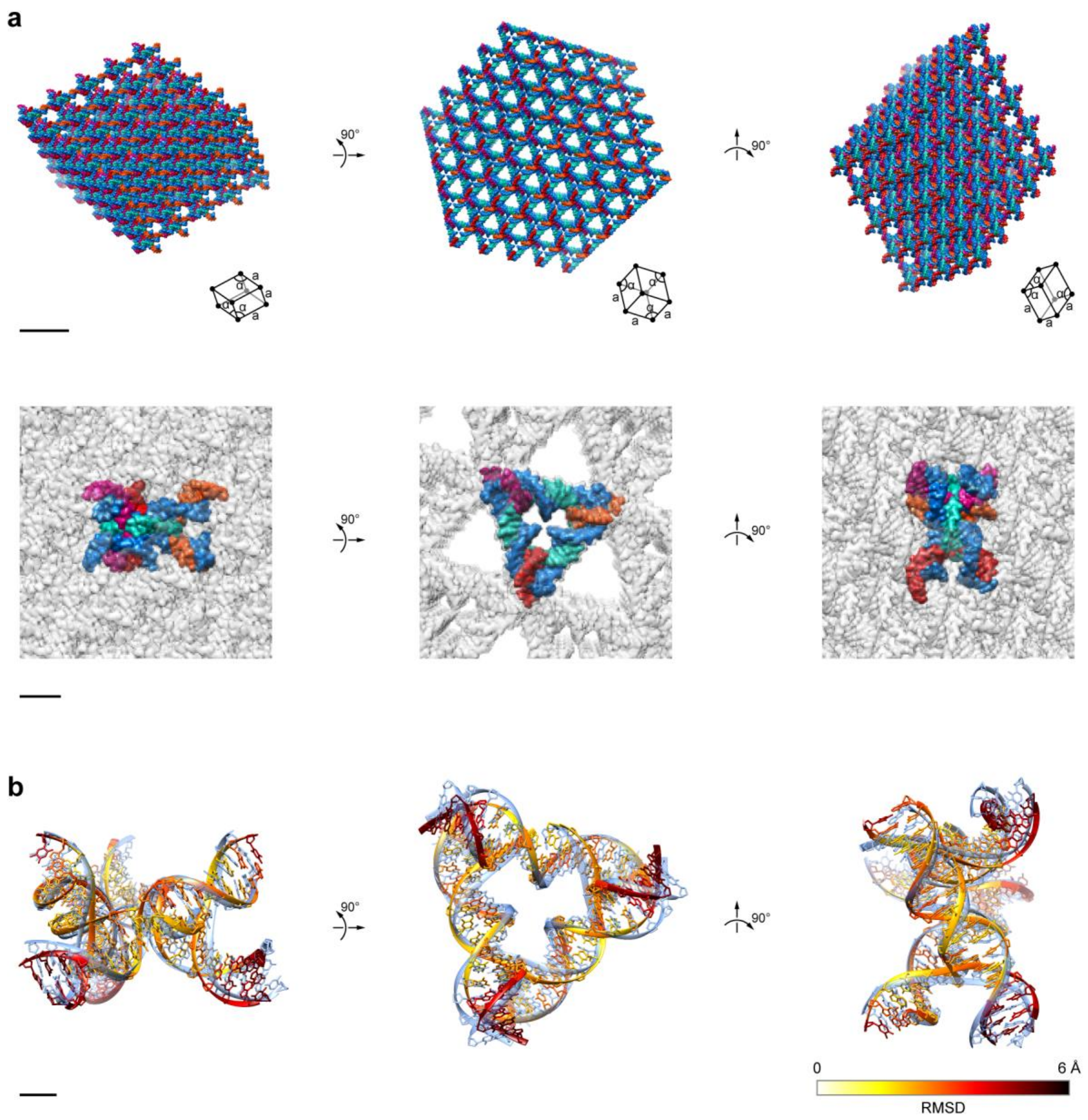

Supplementary Figure 15 | Three orthogonal views of each structure in Fig. 4. (**a**) The predicted crystal structure of PDB ID 3GBI. Scale bars are 10 nm for the overall crystal structure and 2 nm for the zoomed-in views. (**b**) Zoomed-in central single crystal cell aligned with the crystallographic structure (light blue). Scale bar is 1 nm.

a $a = 0.1$, $b = 1$

b $a = 10$, $b = 1$

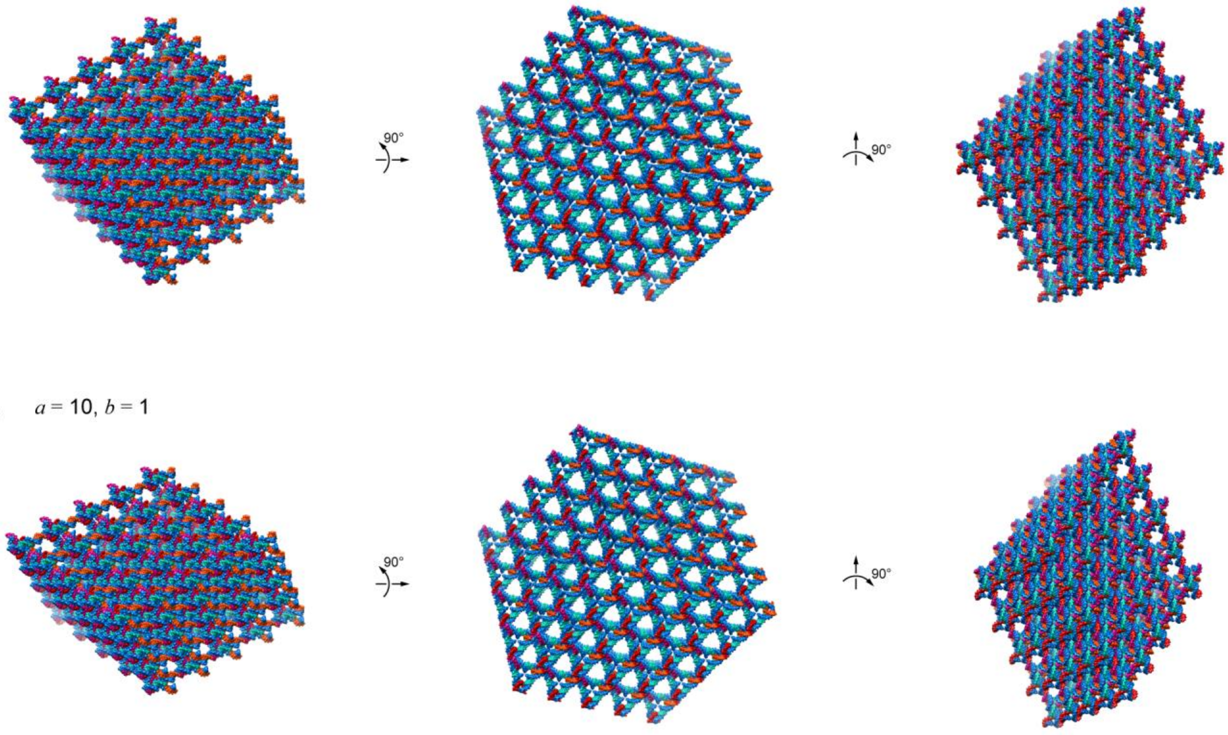

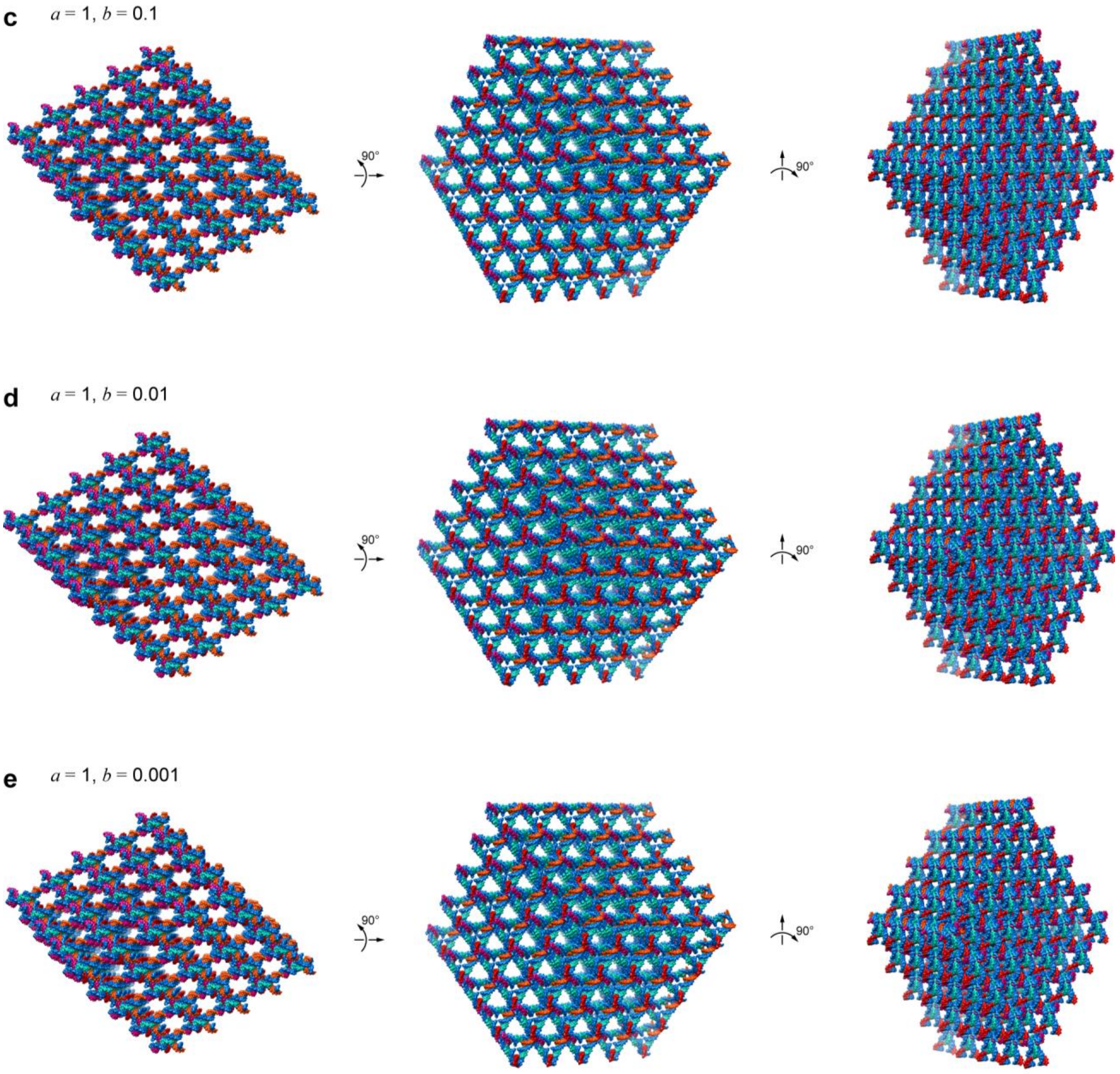

Supplementary Figure 16 | Sensitivity analysis of the 3D lattice crystal. The standard value of rotational junction stiffness k_{twist} = 135.3 pN nm rad^{-1} corresponding to the interhelical angle J_{twist} is multiplied by the factor a = 0.1 (**a**) and 10 (**b**). The standard values of the bend and twist moduli of DNA nicks are multiplied by the factor b = 0.1 (**c**), 0.01 (**d**), and 0.001 (**e**).

a $a = 0.1$, $b = 1$

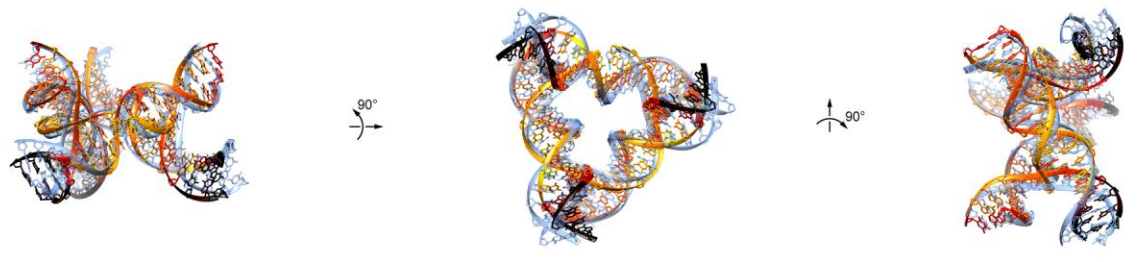

b $a = 10$, $b = 1$

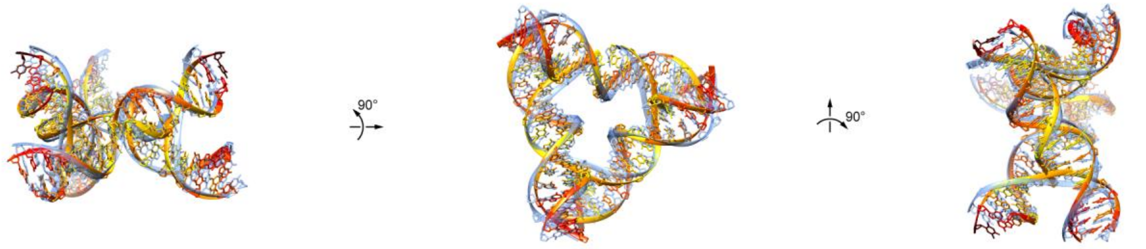

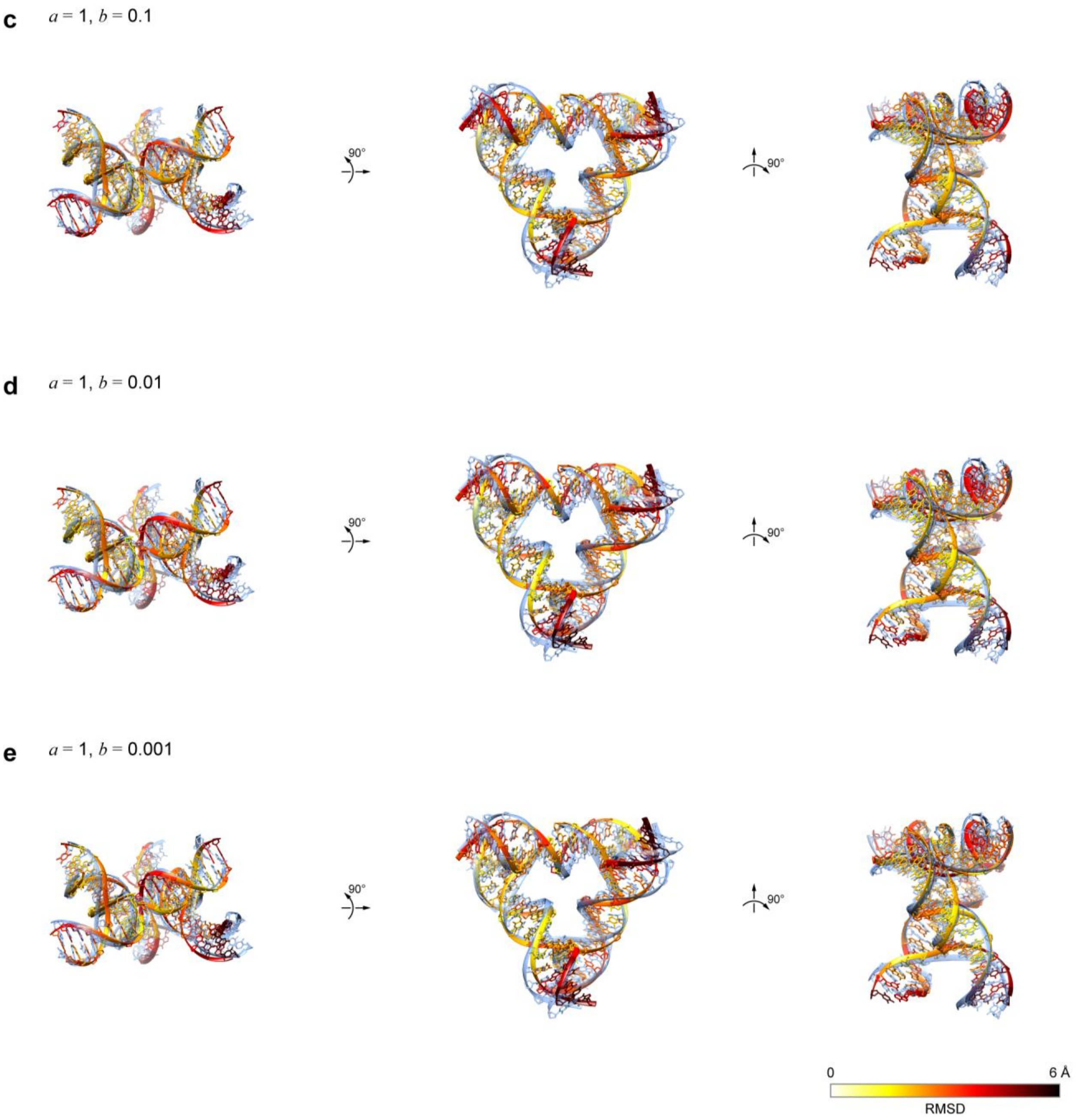

Supplementary Figure 17 | Zoomed-in single crystal cells in the center of the 3D lattice crystals. Each nucleotide is colored according to the RMSD from the crystallographic structure (blue, PDB ID: 3GBI). The standard value of rotational junction stiffness k_{twist} = 135.3 pN nm rad^{-1} corresponding to the interhelical angle J_{twist} is multiplied by the factor a = 0.1 (**a**) and 10 (**b**). The standard values of the bend and twist moduli of DNA nicks are multiplied by the factor b = 0.1 (**c**), 0.01 (**d**), and 0.001 (**e**).

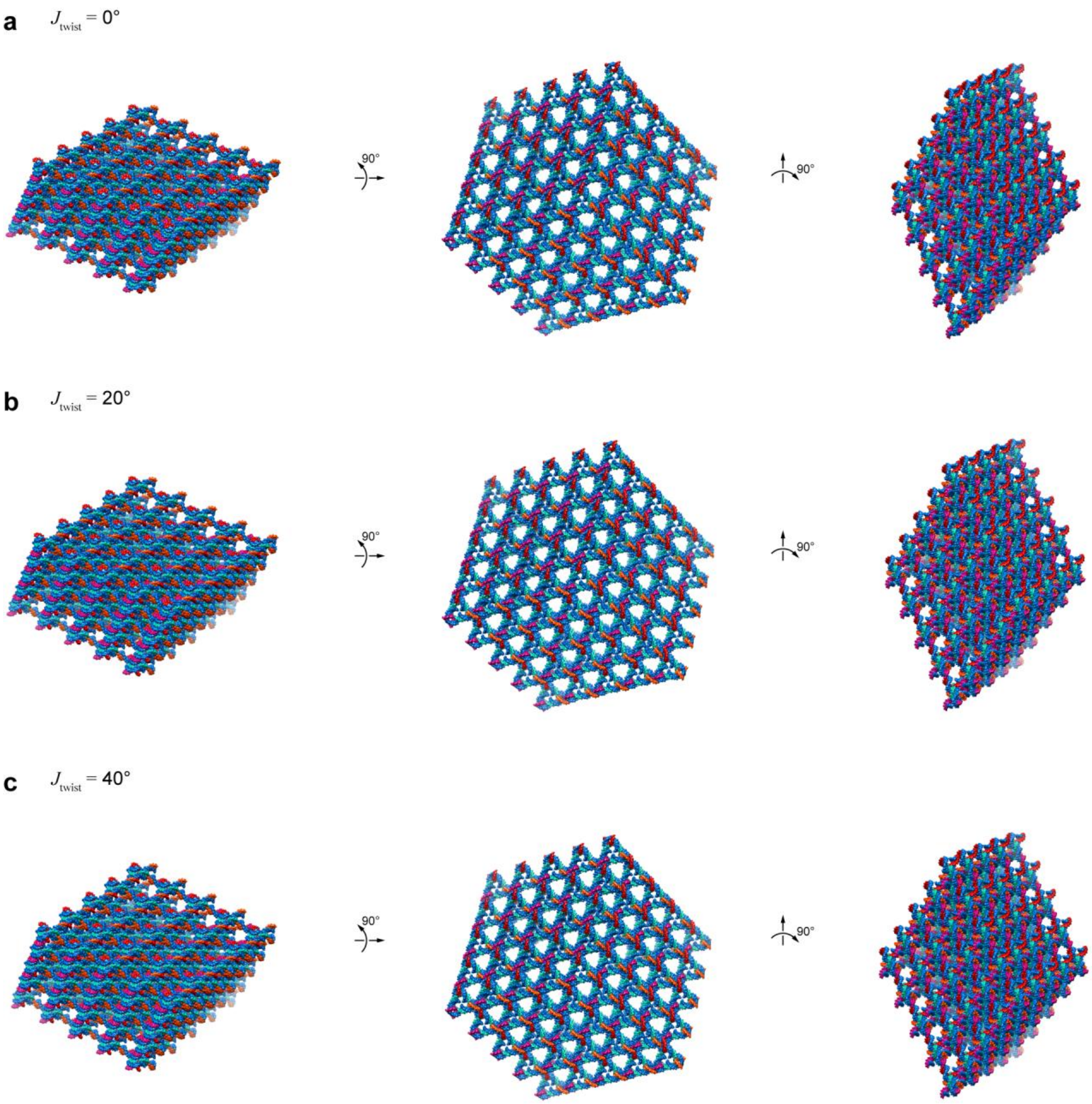

a
J_{twist} = 0°
90°
90°
b
J_{twist} = 20°
90°
90°
c
J_{twist} = 40°
90°
90°

d J_{twist} = 80°

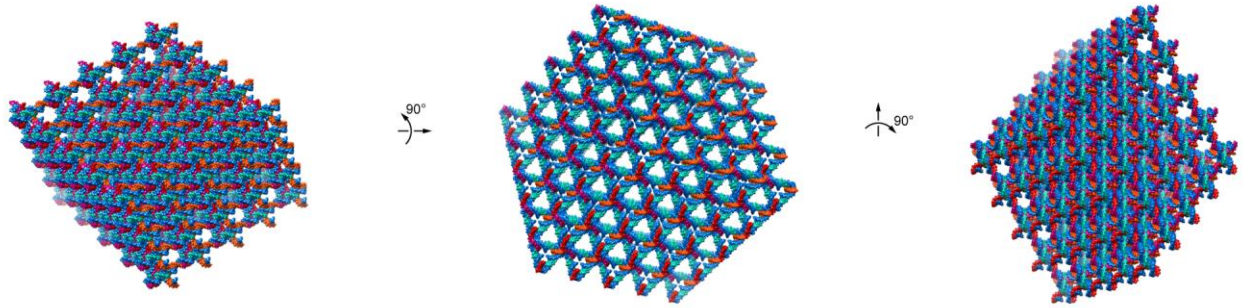

e J_{twist} = 100°

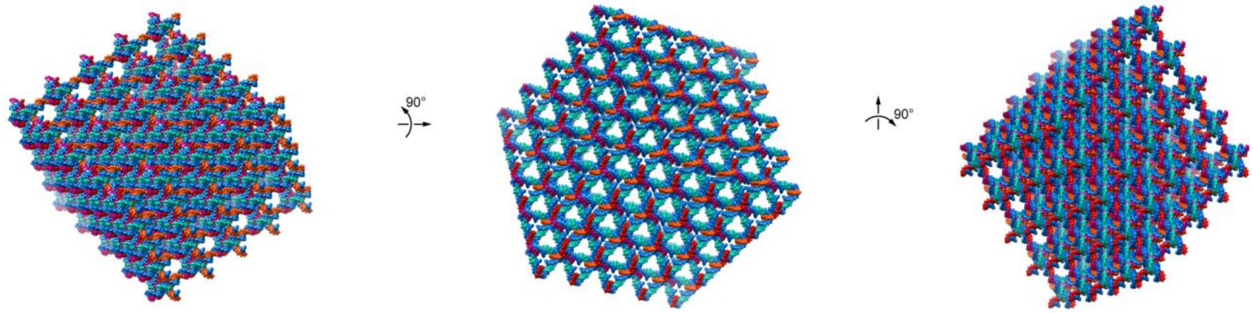

Supplementary Figure 18 | Sensitivity analysis of the 3D lattice crystal. The ground-state value of J_{twist} in four-way junctions equals to 0° (**a**), 20° (**b**), 40° (**c**), 80° (**d**), and 100° (**e**).

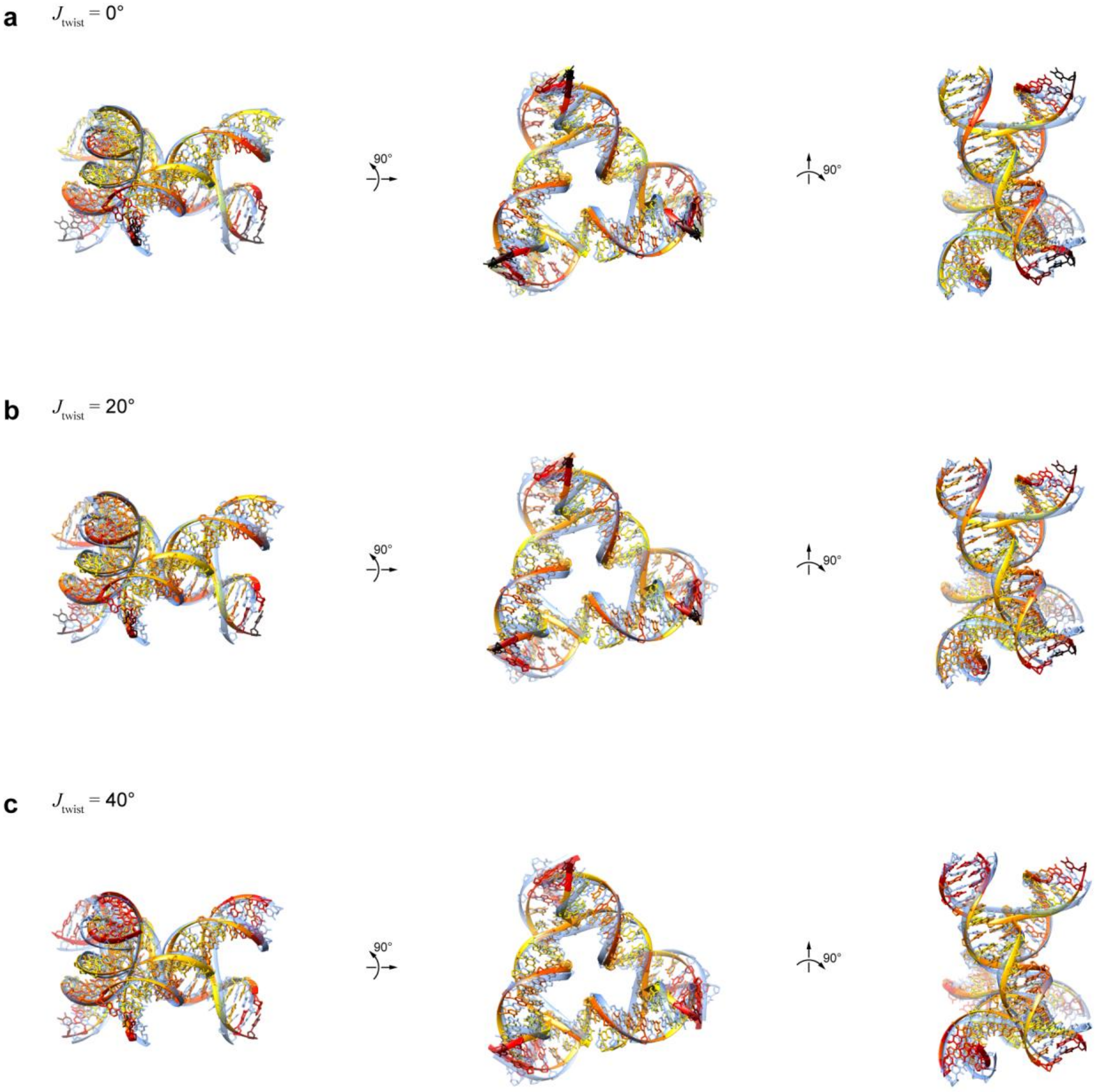
a
J_{twist} = 0°
90°
90°
b
J_{twist} = 20°
90°
90°
c
J_{twist} = 40°
90°
90°

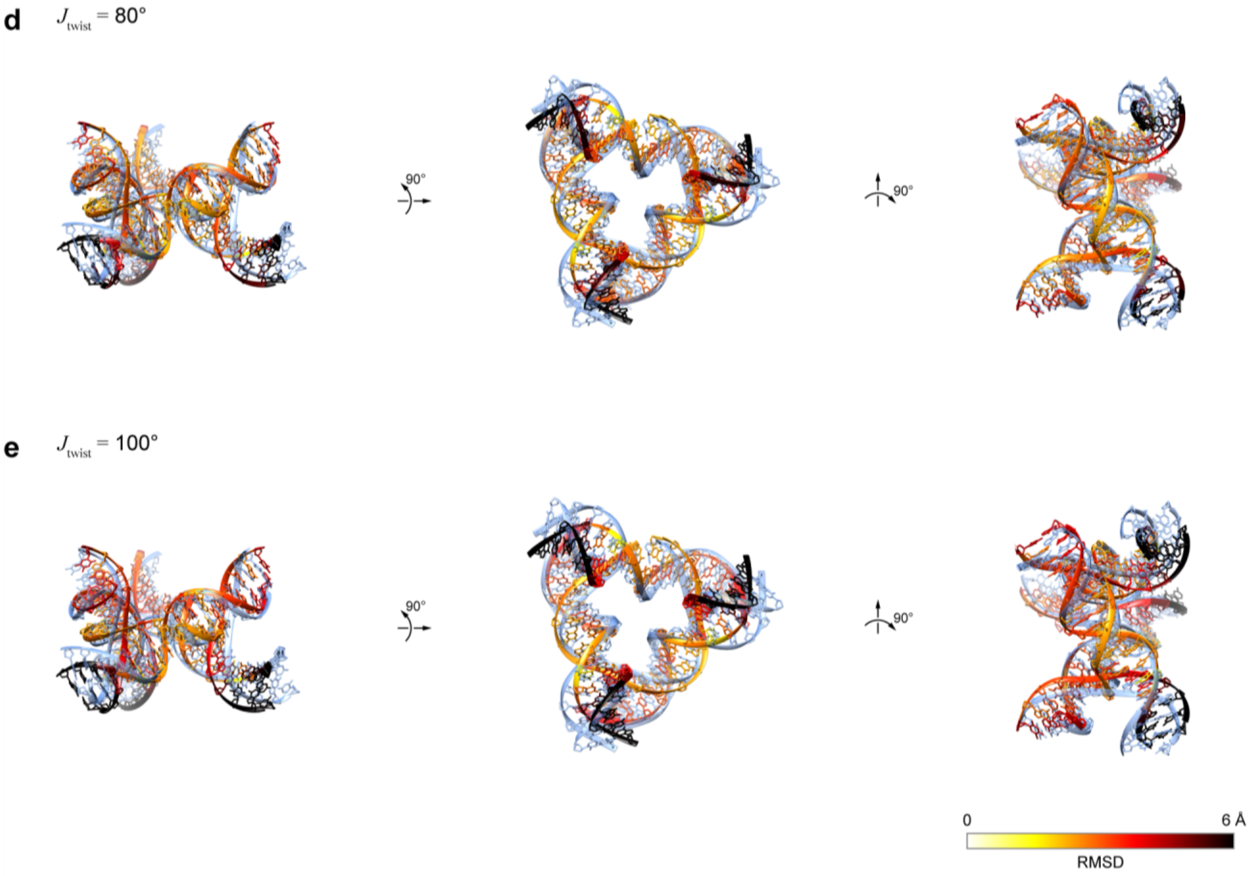

Supplementary Figure 19 | Zoomed-in single crystal cells in the center of the 3D lattice crystals. Each nucleotide is colored according to the RMSD from the crystallographic structure (blue, PDB ID: 3GBI). The ground-state value of J_{twist} in four-way junctions equals to 0° (**a**), 20° (**b**), 40° (**c**), 80° (**d**), and 100° (**e**).

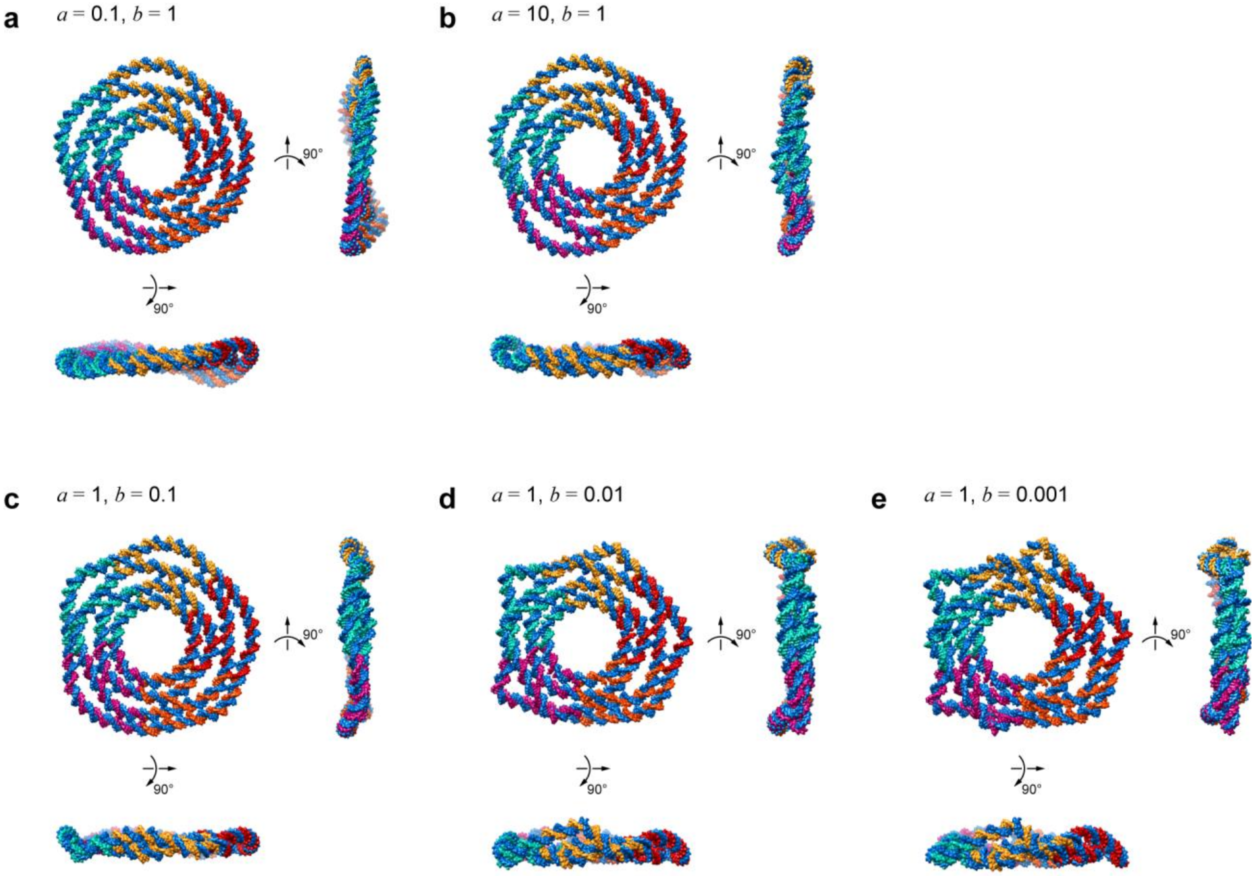

Supplementary Figure 20 | Sensitivity analysis of the 4-layer ring. The standard value of rotational junction stiffness k_{twist} = 135.3 pN nm rad^{-1} corresponding to the interhelical angle J_{twist} is multiplied by the factor a = 0.1 (**a**) and 10 (**b**). The standard values of the bend and twist moduli of DNA nicks are multiplied by the factor b = 0.1 (**c**), 0.01 (**d**), and 0.001 (**e**).

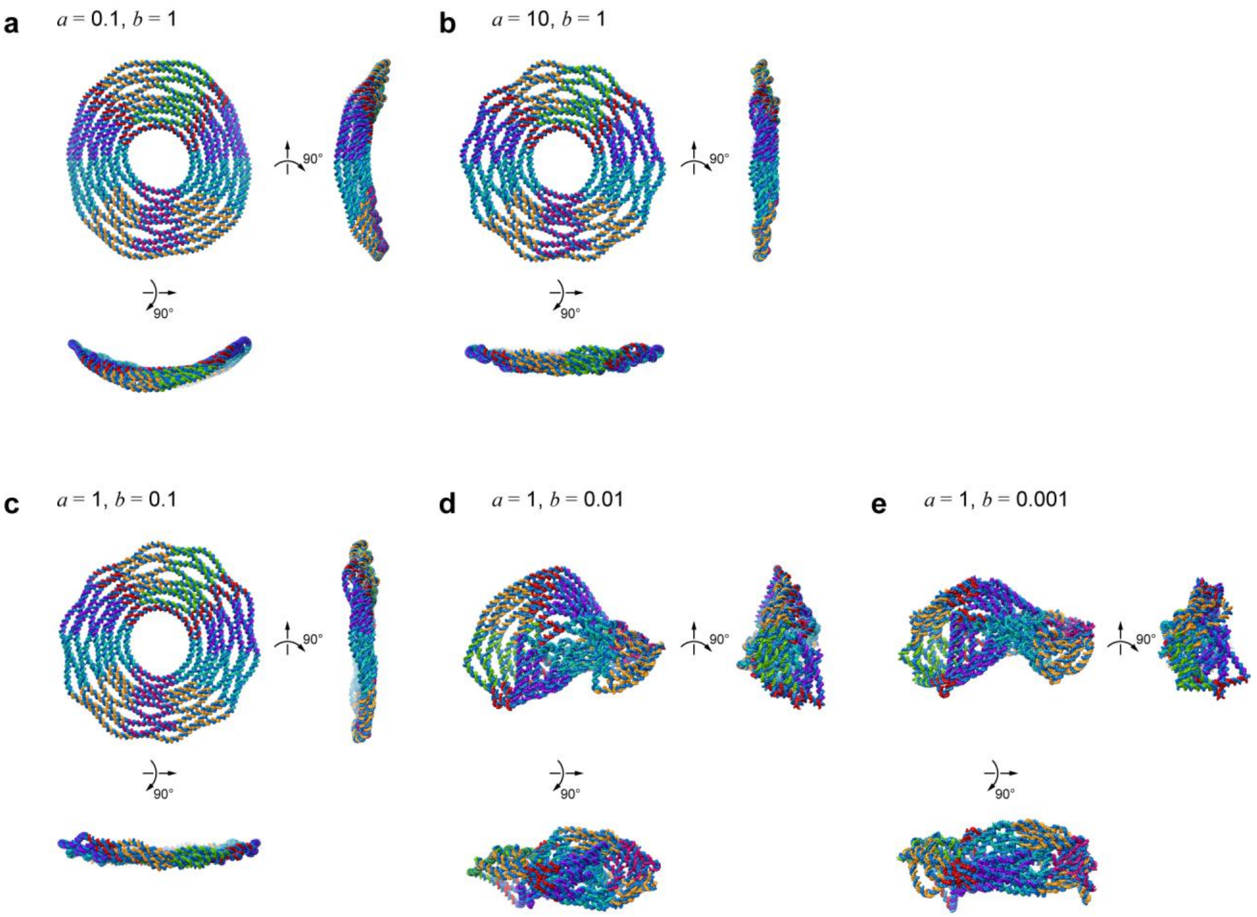

Supplementary Figure 21 | Sensitivity analysis of the 9-layer ring origami. The standard value of rotational junction stiffness k_{twist} = 135.3 pN nm rad^{-1} corresponding to the interhelical angle J_{twist} is multiplied by the factor a = 0.1 (**a**) and 10 (**b**). The standard values of the bend and twist moduli of DNA nicks are multiplied by the factor b = 0.1 (**c**), 0.01 (**d**), and 0.001 (**e**).

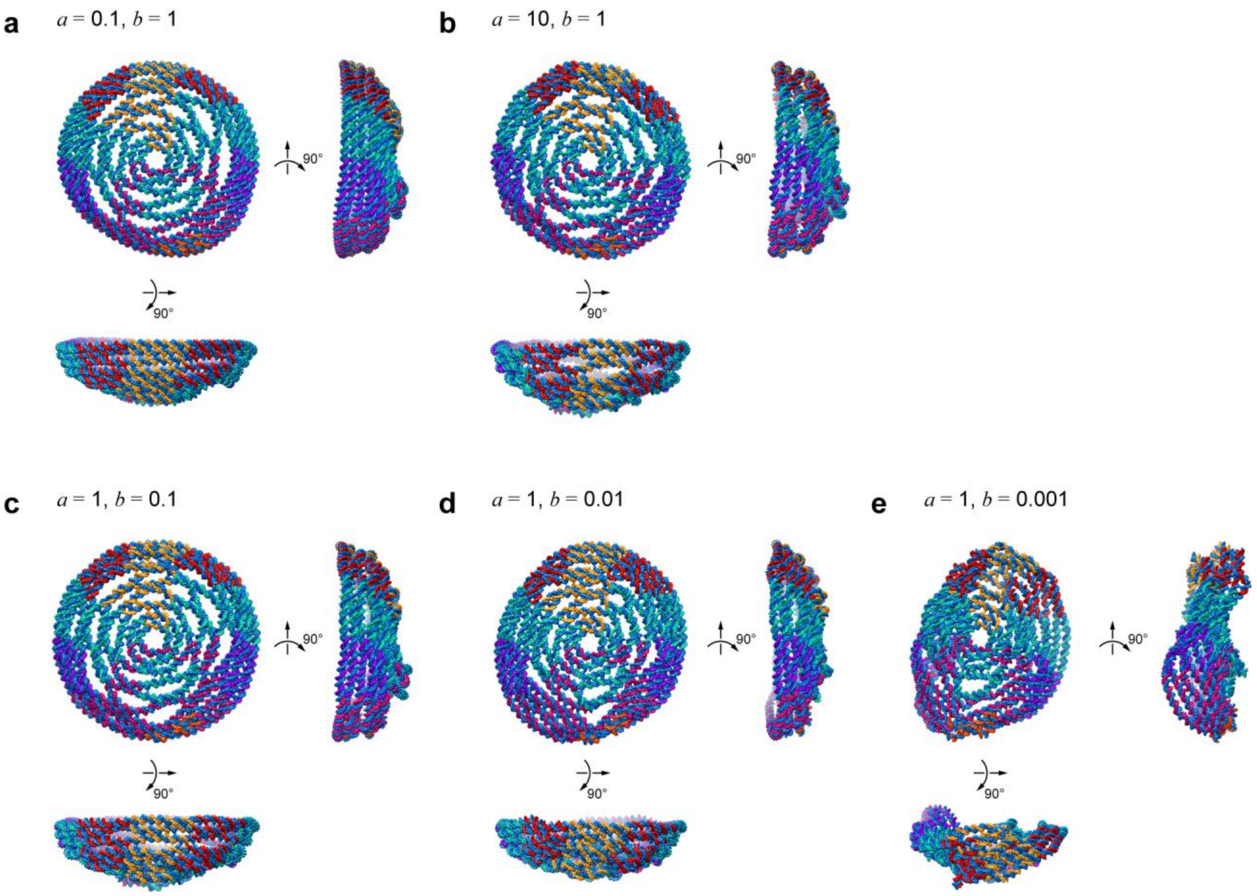

Supplementary Figure 22 | Sensitivity analysis of the 12-layer hemispherical origami. The standard value of rotational junction stiffness k_{twist} = 135.3 pN nm rad^{-1} corresponding to the interhelical angle J_{twist} is multiplied by the factor a = 0.1 (**a**) and 10 (**b**). The standard values of the bend and twist moduli of DNA nicks are multiplied by the factor b = 0.1 (**c**), 0.01 (**d**), and 0.001 (**e**).

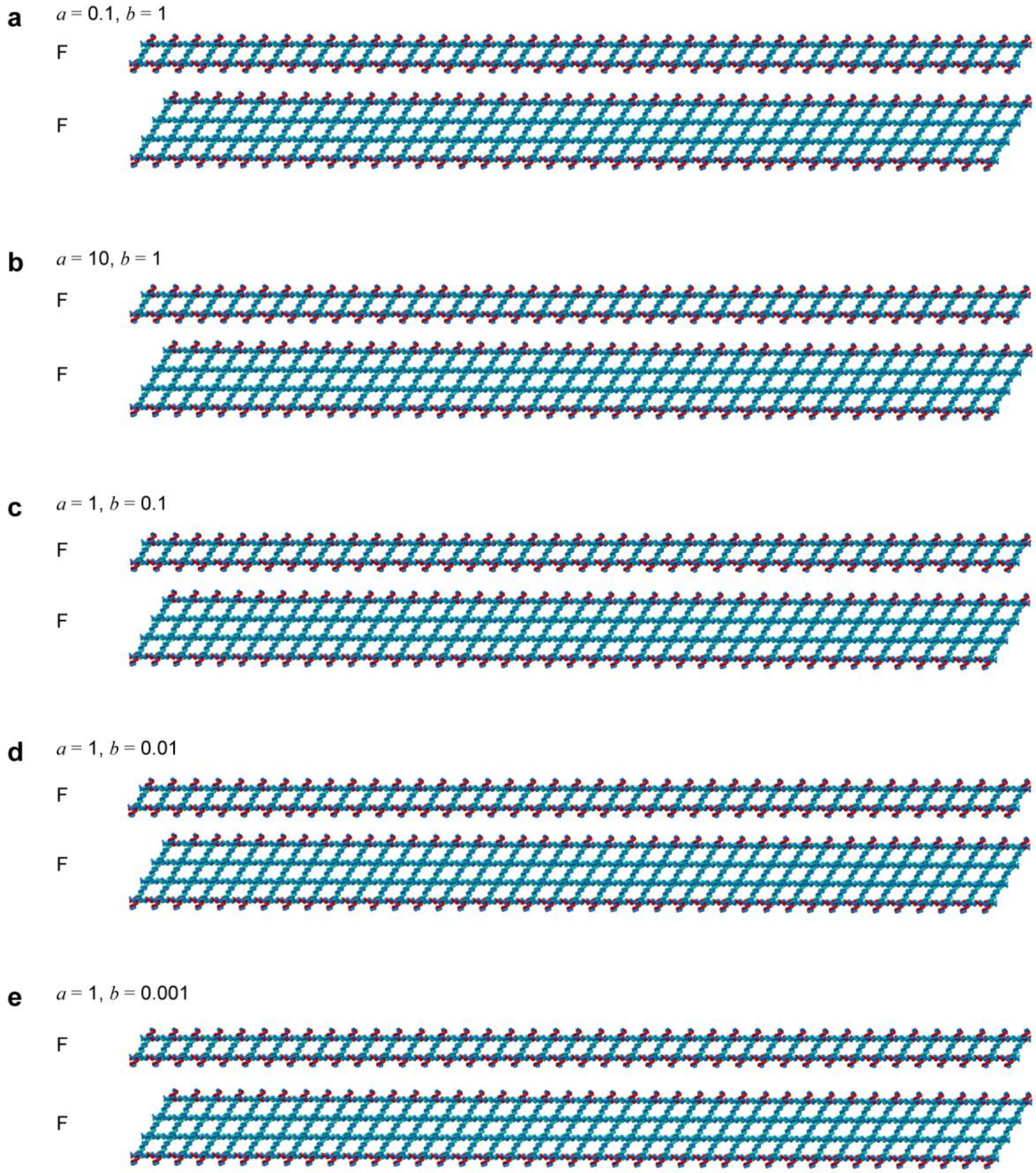

Supplementary Figure 23 | Sensitivity analysis of the (21, 21) lattices. The standard value of rotational junction stiffness k_{twist} = 135.3 pN nm rad^{-1} corresponding to the interhelical angle J_{twist} is multiplied by the factor a = 0.1 (**a**) and 10 (**b**). The standard values of the bend and twist moduli of DNA nicks are multiplied by the factor b = 0.1 (**c**), 0.01 (**d**), and 0.001 (**e**). The 40×2 (upper) and 40×4 (lower) lattices are presented in each panel. All the 3D structures are flat (F), as marked to the left of the 3D structures.

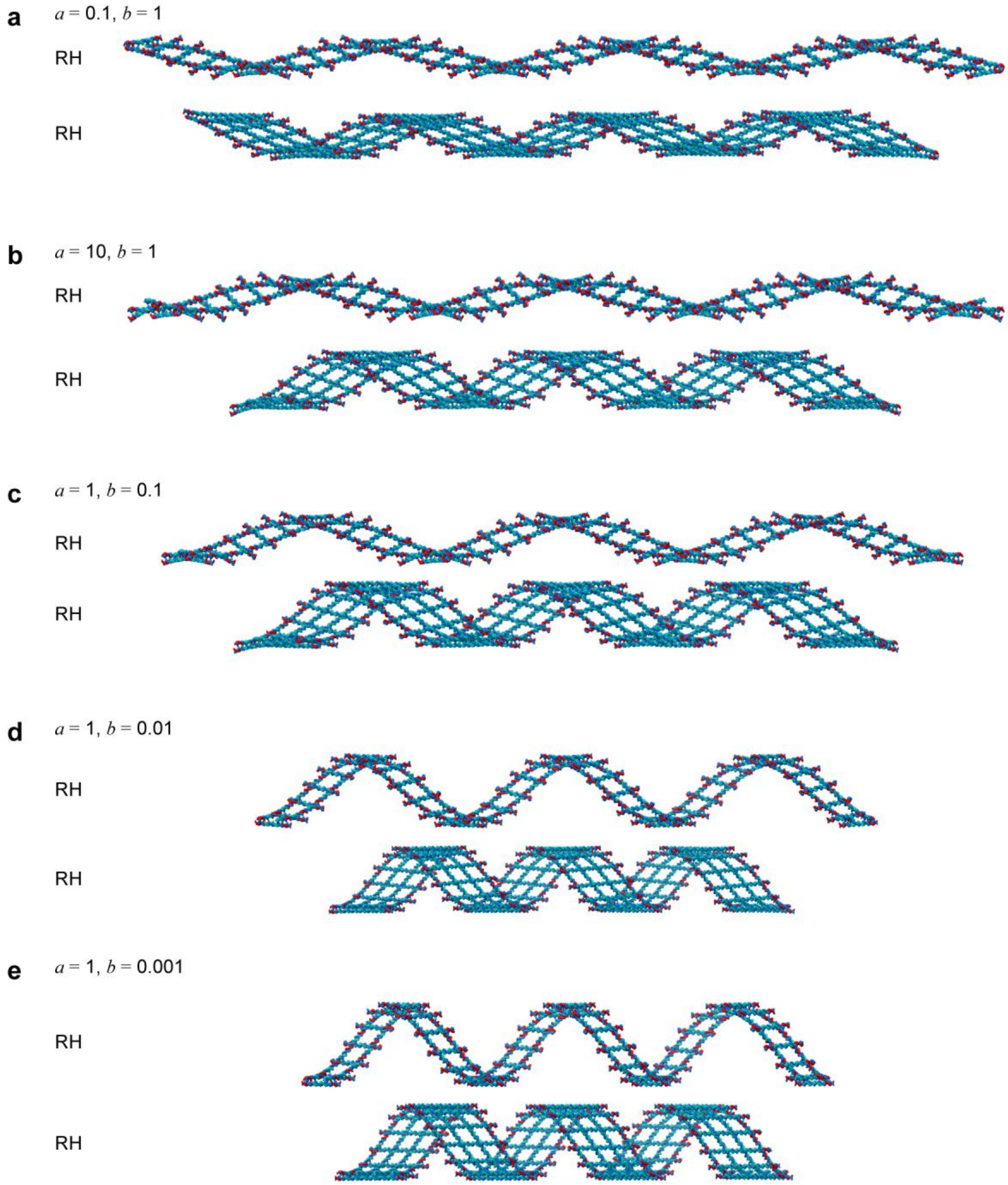

Supplementary Figure 24 | Sensitivity analysis of the (22, 21) lattices. The standard value of rotational junction stiffness k_{twist} = 135.3 pN nm rad^{-1} corresponding to the interhelical angle J_{twist} is multiplied by the factor a = 0.1 (**a**) and 10 (**b**). The standard values of the bend and twist moduli of DNA nicks are multiplied by the factor b = 0.1 (**c**), 0.01 (**d**), and 0.001 (**e**). The 40×2 (upper) and 40×4 (lower) lattices are presented in each panel. All the 3D structures are right-handed (RH), as marked to the left of the 3D structures.

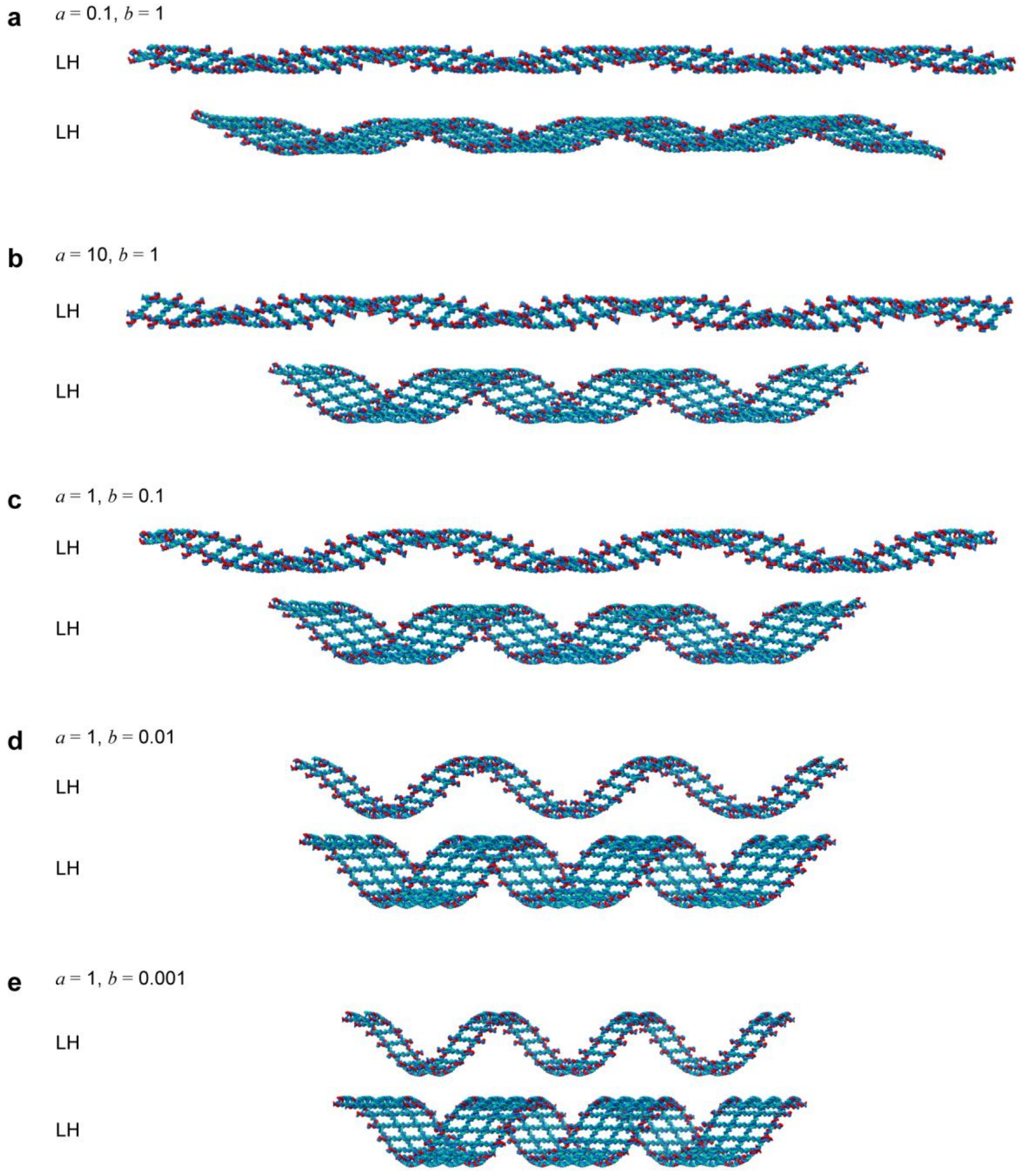

Supplementary Figure 25 | Sensitivity analysis of the (20, 21) lattices. The standard value of rotational junction stiffness k_{twist} = 135.3 pN nm rad^{-1} corresponding to the interhelical angle J_{twist} is multiplied by the factor a = 0.1 (**a**) and 10 (**b**). The standard values of the bend and twist moduli of DNA nicks are multiplied by the factor b = 0.1 (**c**), 0.01 (**d**), and 0.001 (**e**). The 40×2 (upper) and 40×4 (lower) lattices are presented in each panel. All the 3D structures are left-handed (LH), as marked to the left of the 3D structures.

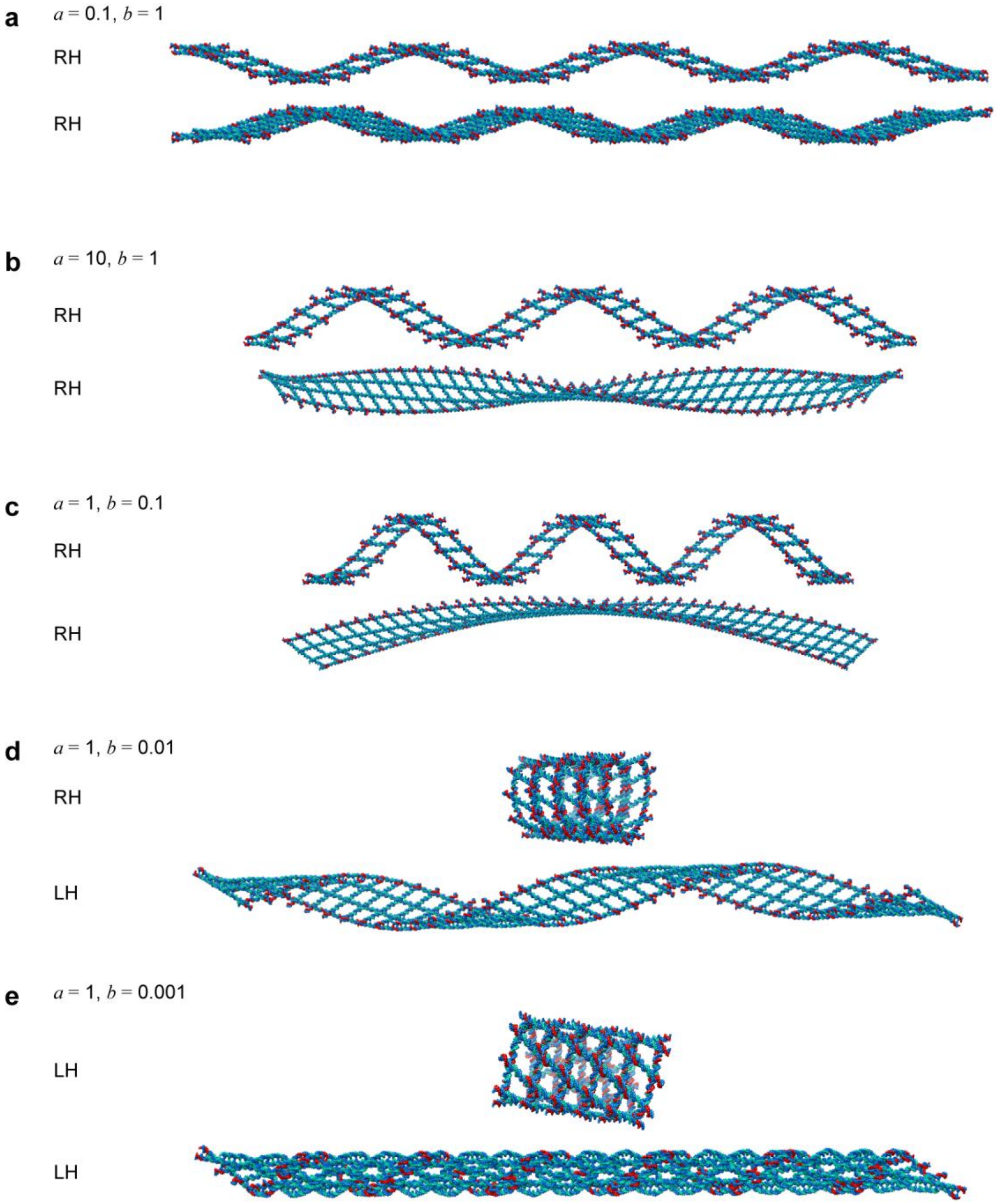

Supplementary Figure 26 | Sensitivity analysis of the (22, 22) lattices. The standard value of rotational junction stiffness k_{twist} = 135.3 pN nm rad^{-1} corresponding to the interhelical angle J_{twist} is multiplied by the factor a = 0.1 (**a**) and 10 (**b**). The standard values of the bend and twist moduli of DNA nicks are multiplied by the factor b = 0.1 (**c**), 0.01 (**d**), and 0.001 (**e**). The 40×2 (upper) and 40×4 (lower) lattices are presented in each panel. As the bend and twist moduli of DNA nicks decrease, the chirality of the 40×2 and 40×4 lattices gradually changes from right-handed (RH) to left-handed (LH).

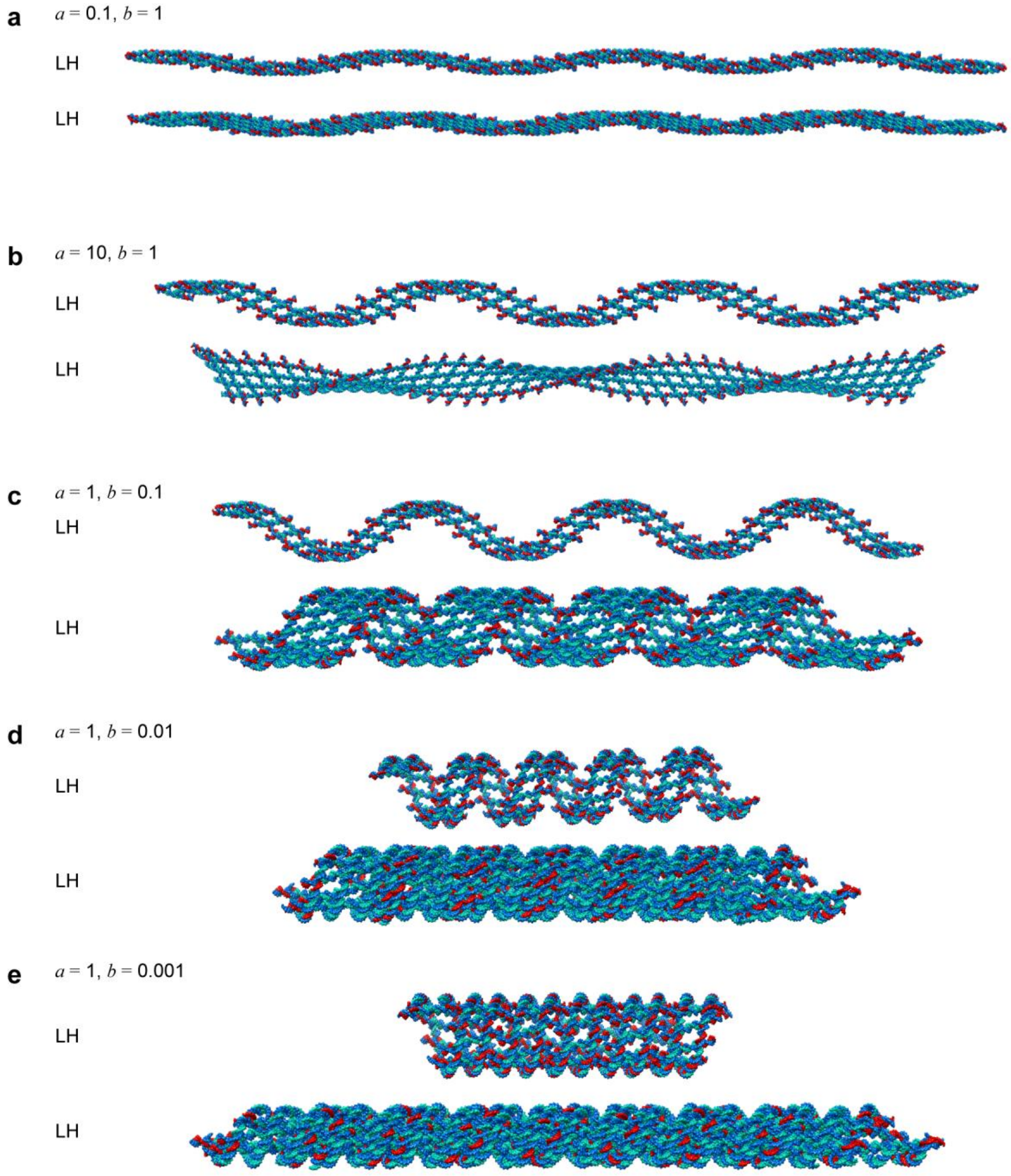

Supplementary Figure 27 | Sensitivity analysis of the (20, 20) lattices. The standard value of rotational junction stiffness k_{twist} = 135.3 pN nm rad^{-1} corresponding to the interhelical angle J_{twist} is multiplied by the factor a = 0.1 (**a**) and 10 (**b**). The standard values of the bend and twist moduli of DNA nicks are multiplied by the factor b = 0.1 (**c**), 0.01 (**d**), and 0.001 (**e**). The 40×2 (upper) and 40×4 (lower) lattices are presented in each panel. All the 3D structures are left-handed (LH), as marked to the left of the 3D structures.

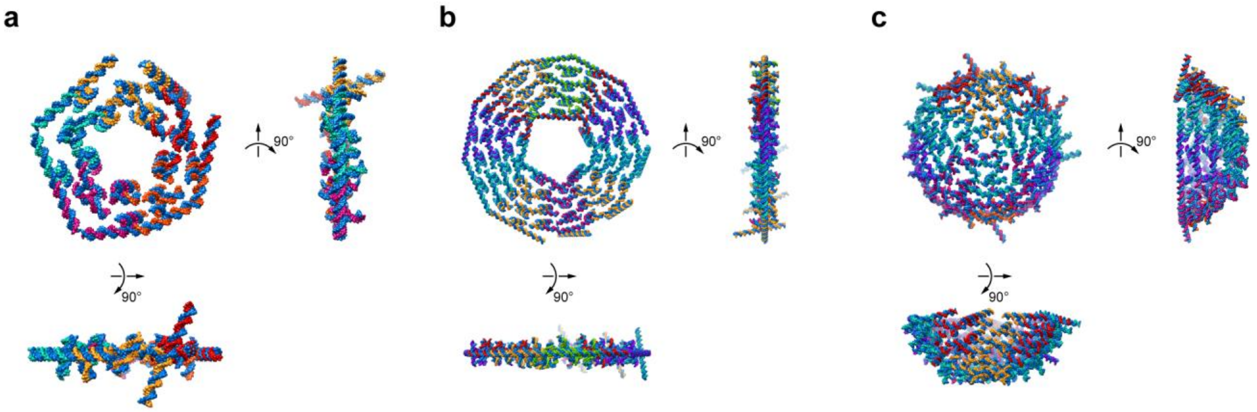

Supplementary Figure 28 | Initial configurations in three orthogonal views of the concentric ring structures in Fig. 2. (**a**) The 4-layer ring, (**b**) the 9-layer ring origami, and (**c**) the 12-layer hemispherical origami.

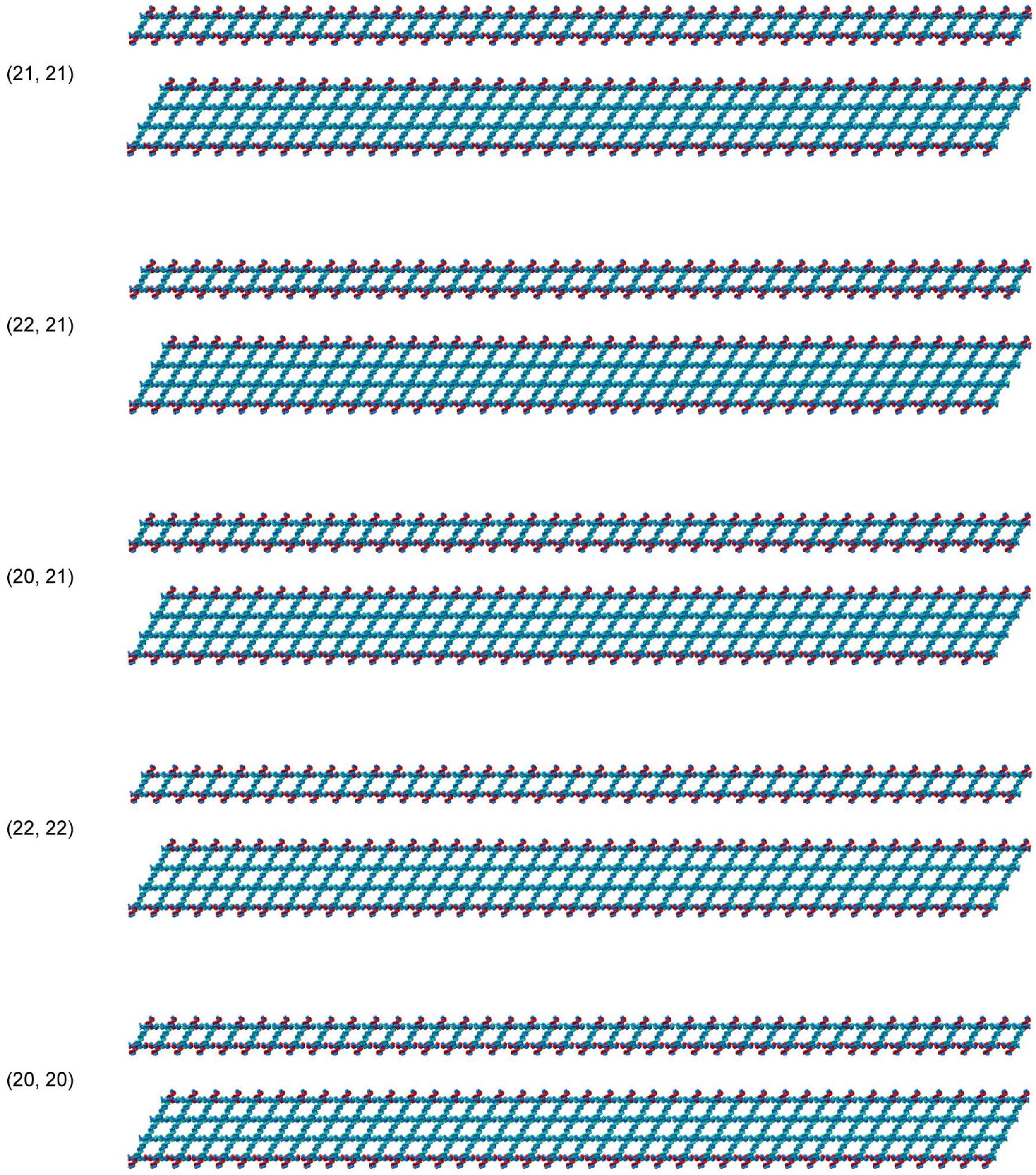

Supplementary Figure 29 | Initial configurations of the DNA ribbon structures consisting of 40×2 and 40×4 immobile four-way junctions in Fig. 3 and Supplementary Fig. 13. Junctions are separated in x- and y-direction by (n_x, n_y) = (21, 21), (22, 21), (20, 21), (22, 22), and (20, 20) bps.

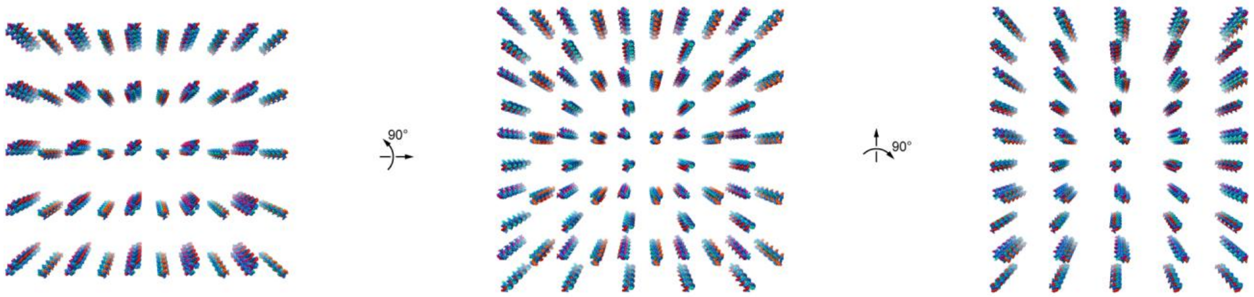

Supplementary Figure 30 | Initial configuration in three orthogonal views of the 3D crystal lattice in Fig. 4.

Supplementary Tables

Supplementary Table 1 | Summary of geometric and mechanical parameter values of the "stacked-X" four-way junction.

**Four-way junction parameters**	**Parameter Values**
Ground-state interhelical distance s [nm]	1.85
Ground-state interhelical angle J_{twist} [°]	60
Translational stiffness coefficient k_{t1} along axis $\mathbf{e}_1$ [pN nm^{-1}]	6.47×10^{6}
Translational stiffness coefficient k_{t2} along axis $\mathbf{e}_2$ [pN nm^{-1}]	6.47×10^{6}
Translational stiffness coefficient k_{t3} along axis $\mathbf{e}_3$ [pN nm^{-1}]	6.47×10^{6}
Rotational stiffness coefficient k_{r1} about axis $\mathbf{e}_1$ [pN nm rad^{-1}]	1,353
Rotational stiffness coefficient k_{r2} about axis $\mathbf{e}_2$ [pN nm rad^{-1}]	1,353
Rotational stiffness coefficient k_{twist} about axis $\mathbf{e}_3$ [pN nm rad^{-1}]	135.3

Supplementary Table 2 | RMSD in nm of the all-atom 3D structures in panels (a-e) of Supplementary Figs. 20-27 calculated using various k_{twist} and nick stiffness values from the corresponding ones using standard parameter values. RMSD values before and after slashes are those of 40×2 and 40×4 lattices, respectively.

RMSD (nm)	Panel a	Panel b	Panel c	Panel d	Panel e
**Supplementary Fig. 20**	0.83	0.55	0.38	1.36	1.75
**Supplementary Fig. 21**	2.68	0.98	0.91	14.85	16.35
**Supplementary Fig. 22**	0.99	0.65	0.56	1.86	6.23
**Supplementary Fig. 23**	0.00 / 0.00	0.00 / 0.00	0.00 / 0.00	0.00 / 0.00	0.00 / 0.00
**Supplementary Fig. 24**	2.04 / 8.51	0.28 / 1.24	3.76 / 5.22	20.26 / 18.53	31.20 / 24.85
**Supplementary Fig. 25**	1.74 / 11.75	0.27 / 2.17	3.92 / 4.90	21.14 / 15.03	29.30 / 18.74
**Supplementary Fig. 26**	10.47 / 9.88	2.87 / 2.31	14.11 / 12.18	64.35 / 13.01	65.07 / 27.61
**Supplementary Fig. 27**	6.14 / 22.59	1.76 / 24.99	12.58 / 15.90	46.09 / 29.71	53.85 / 23.76

Supplementary Notes

Supplementary Note 1 Graph traversal algorithm

A generic programmed DNA assembly is modeled as a directed graph (Supplementary Figs. 1 and 2), which is subsequently processed in order to define the downstream finite element model. The graph traversal algorithm comprises the following steps.

Input. The sequence and topology information as a directed graph model. Each node represents a nucleotide with initial Cartesian coordinates along with a unique nucleotide ID.

Step 1. Identify all DNA strands.

Step 1a. *Set all nucleotides as unvisited.*

Step 1b. *If all nucleotides in the input are visited, then go to Step 2.*

Step 1c. *Find the unvisited nucleotide with the lowest nucleotide ID.*

Step 1d. *Find the nucleotide sequence of the current strand. Set all nucleotides in the current strand as visited. Assign the current strand a unique strand ID.*

Step 1e. *Go to Step 1b.*

Step 2. Identify all multi-way junctions.

Step 2a. *Find all branch points connecting two DNA duplexes at junctions. Branch points are defined as graph nodes where the 3′-neighbor is contained in a different duplex.*

Step 2b. *Assign each junction a unique junction ID and a set of branch points. For example, a four-way junction is assigned four branch points corresponding to the four duplex arms.*

Step 2c. *Assign each branch point a unique arm ID.*

Step 2d. *Specify the initial position and orientation of each junction. If a junction is four-way, determine its isomeric state using the initial Cartesian coordinates of the nucleotides.*

Step 3. Identify topological connectivity between multi-way junctions and duplex arm lengths.

Step 3a. *Set all branch points as unvisited.*

Step 3b. *If all branch points are visited, then go to Step 4.*

Step 3c. *Find an unvisited branch point A and mark it as visited. Search for a branch point B that connects to A via a duplex. If found, mark B as visited.*

Step 3d. *Determine lengths of duplex arms corresponding to branch points A and B.*

Step 3e. *Go to Step 3b.*

Step 4. Identify all nicks in duplex arms found in Step 3 along with all unpaired nucleotides.

Output. The set of interconnected multi-way junctions with associated connectivities and lengths of duplex arms.

Supplementary Note 2 Finite element model definition

DNA duplex model

As shown in Supplementary Fig. 3, Duplexes are modeled as linear elastic beams with stretch modulus S_{dsDNA} = 1,100 pN, bend modulus B_{dsDNA} = 230 pN nm^2, and twist modulus T_{dsDNA} = 460 pN nm^2 [2, 3]. Each basepair (bp) is represented as a finite element node with axial rise per bp L =

0.34 nm whose center and orientation are defined using the 3DNA convention. Nicks are modeled as previously performed, using reduced twisting and bending stiffnesses[3].

Generic multi-way junction model

Multi-way junctions in DNA nanostructures are treated as a set of duplexes interconnected by phosphate-sugar backbones that constrain their relative ground-state angles with harmonic forces and moments associated with their relative translational and rotational degrees of freedom (DOFs). The phosphate-sugar backbone connecting two bps in different duplexes is modeled using an alignment element connecting two finite element nodes, denoted by n_b and n_t, corresponding to the same two bps (Supplementary Fig. 4). Thus, the alignment element controls the relative position and orientation of node n_t with respect to node n_b. Applying this model specifically to junctions, the alignment element connecting two duplexes in a single junction requires the following input parameters: two right-handed A-triads rigidly attached to nodes n_b and n_t, two right-handed B-triads rigidly connected to n_b and n_t, and six stiffness coefficients used to calculate restorative forces and moments. This modeling procedure is easily generalizable to three-way, five-way, and other multi-way junctions with arbitrarily specified ground-state angles and associated flexibilities that are known empirically.

The first step to create an alignment element is to define its associated triads. In Supplementary Fig. 4, the A-triad rigidly attached to node n_b has the center $\mathbf{a}_{b0}$ at n_b and three axes $\mathbf{a}_{b1}$, $\mathbf{a}_{b2}$, and $\mathbf{a}_{b3}$. Vectors $\mathbf{a}_{b0}$, $\mathbf{a}_{b1}$, $\mathbf{a}_{b2}$, and $\mathbf{a}_{b3}$ are defined in the global reference frame. Similarly, the B-triad rigidly connected to node n_b has the center $\hat{\mathbf{b}}_{b0}$ and three axes $\hat{\mathbf{b}}_{b1}$, $\hat{\mathbf{b}}_{b2}$, and $\hat{\mathbf{b}}_{b3}$. Note that vectors $\hat{\mathbf{b}}_{b0}$, $\hat{\mathbf{b}}_{b1}$, $\hat{\mathbf{b}}_{b2}$, and $\hat{\mathbf{b}}_{b3}$ are defined in the A-triad of node n_b. Thus, represented in the global reference frame, the B-triad of node n_b has the center,

$$\mathbf{b}_{b0} = \mathbf{a}_{b0} + \begin{pmatrix}\mathbf{a}_{b1} & \mathbf{a}_{b2} & \mathbf{a}_{b3}\end{pmatrix}\hat{\mathbf{b}}_{b0} \tag{1}$$

where $\begin{pmatrix}\mathbf{a}_{b1} & \mathbf{a}_{b2} & \mathbf{a}_{b3}\end{pmatrix}$ is a matrix with $\mathbf{a}_{b1}$, $\mathbf{a}_{b2}$, $\mathbf{a}_{b3}$ as three columns, and three axes $\mathbf{b}_{b1}$, $\mathbf{b}_{b2}$, and $\mathbf{b}_{b3}$ given by,

$$\begin{pmatrix}\mathbf{b}_{b1} & \mathbf{b}_{b2} & \mathbf{b}_{b3}\end{pmatrix} = \begin{pmatrix}\mathbf{a}_{b1} & \mathbf{a}_{b2} & \mathbf{a}_{b3}\end{pmatrix}\begin{pmatrix}\hat{\mathbf{b}}_{b1} & \hat{\mathbf{b}}_{b2} & \hat{\mathbf{b}}_{b3}\end{pmatrix} \tag{2}$$

.

In the same way, the A-triad of node n_t has the center $\mathbf{a}_{t0}$ at n_t and axes $\mathbf{a}_{t1}$, $\mathbf{a}_{t2}$, and $\mathbf{a}_{t3}$ defined in the global reference frame. The B-triad of node n_t has the center $\hat{\mathbf{b}}_{t0}$ and axes $\hat{\mathbf{b}}_{t1}$, $\hat{\mathbf{b}}_{t2}$, and $\hat{\mathbf{b}}_{t3}$ defined in the A-triad of node n_t and the center,

$$\mathbf{b}_{t0} = \mathbf{a}_{t0} + \begin{pmatrix}\mathbf{a}_{t1} & \mathbf{a}_{t2} & \mathbf{a}_{t3}\end{pmatrix}\hat{\mathbf{b}}_{t0} \tag{3}$$

and axes,

$$\begin{pmatrix}\mathbf{b}_{t1} & \mathbf{b}_{t2} & \mathbf{b}_{t3}\end{pmatrix} = \begin{pmatrix}\mathbf{a}_{t1} & \mathbf{a}_{t2} & \mathbf{a}_{t3}\end{pmatrix}\begin{pmatrix}\hat{\mathbf{b}}_{t1} & \hat{\mathbf{b}}_{t2} & \hat{\mathbf{b}}_{t3}\end{pmatrix} \tag{4}$$

represented in the global reference frame.

Once the A- and B-triads of each node are uniquely determined, the misalignments between the B-triads of nodes n_b and n_t, as well as the restorative forces and moments, are calculated in the procedure described as follows.

Translational misalignments along the axes $\mathbf{b}_{b1}$, $\mathbf{b}_{b2}$, and $\mathbf{b}_{b3}$ are,

$$\begin{aligned} r_1 &= \left(\mathbf{b}_{t0} - \mathbf{b}_{b0}\right) \cdot \mathbf{b}_{b1} \\ r_2 &= \left(\mathbf{b}_{t0} - \mathbf{b}_{b0}\right) \cdot \mathbf{b}_{b2} \\ r_3 &= \left(\mathbf{b}_{t0} - \mathbf{b}_{b0}\right) \cdot \mathbf{b}_{b3}. \end{aligned} \tag{5}$$

Rotational misalignments about the axes $\mathbf{b}_{b1}$, $\mathbf{b}_{b2}$, and $\mathbf{b}_{b3}$ can be represented by the following rotation matrix,

$$\mathbf{R} = \begin{pmatrix} \mathbf{b}_{b1} \cdot \mathbf{b}_{t1} & \mathbf{b}_{b1} \cdot \mathbf{b}_{t2} & \mathbf{b}_{b1} \cdot \mathbf{b}_{t3} \\ \mathbf{b}_{b2} \cdot \mathbf{b}_{t1} & \mathbf{b}_{b2} \cdot \mathbf{b}_{t2} & \mathbf{b}_{b2} \cdot \mathbf{b}_{t3} \\ \mathbf{b}_{b3} \cdot \mathbf{b}_{t1} & \mathbf{b}_{b3} \cdot \mathbf{b}_{t2} & \mathbf{b}_{b3} \cdot \mathbf{b}_{t3} \end{pmatrix} \tag{6}$$

which can be converted to axis-angle representation using the rotation angle θ and the rotation axis $\mathbf{u} = (u_1, u_2, u_3)^T$ with respect to the reference frame. Then, three rotational misalignments are given by,

$$\begin{aligned} \theta_1 &= \theta u_1 \\ \theta_2 &= \theta u_2 \\ \theta_3 &= \theta u_3. \end{aligned} \tag{7}$$

Misalignments induce restorative forces and moments in the alignment element that can be written as,

$$\begin{aligned} F_1 &= k_{t1} r_1 \\ F_2 &= k_{t2} r_2 \\ F_3 &= k_{t3} r_3 \\ M_1 &= k_{r1} \theta_1 \\ M_2 &= k_{r2} \theta_2 \\ M_3 &= k_{r3} \theta_3 \end{aligned} \tag{8}$$

where k_{t1}, k_{t2}, and k_{t3}, and k_{r1}, k_{r2}, and k_{r3} are translational and rotational junction stiffness coefficients, respectively, assumed here to be constant.

The finite element model associated with each junction initially resides in its mechanical ground-state zero-energy configuration, defined at an initial position and orientation that must defined empirically. For example, for the four-way junction studied here, we refer to Supplementary Note 4. Application to other multi-way junctions may be explored in future work.

Supplementary Note 3 Calculation of 3D structure

Having defined the finite element model of DNA duplexes and multi-way junctions, we next perform an energy minimization procedure in which the equilibrium 3D structure of the programmed DNA assembly is computed. For this purpose we employ the commercial finite element analysis program ADINA Version 8.9 (ADINA R&D, Watertown, MA, USA). The alignment element is used to displace the two finite element nodes, denoted by n_b and n_t, corresponding to the two bps located at the ends of two duplexes in order for them to coincide

with each other without any translational and rotational mismatch. While this procedure is completely general in that it only requires the topological connectivity of neighboring junctions to be known, we focus application of the procedure here to four-way junctions that reside in a single geometric ground-state where the value of the scissor-like interhelical angle J_{twist} is known empirically (Fig. 1).

As shown in Supplementary Fig. 5, the specific alignment element rigidly attaches two B-triads to n_b and n_t, respectively. The centers $\mathbf{b}_{b0}$ and $\mathbf{b}_{t0}$ coincide with n_b and n_t, respectively. Axes $\mathbf{b}_{b1}$ and $\mathbf{b}_{t1}$ are defined to be coincident with the duplex axes, and the other axes are determined using the geometry of 10.5 bp per turn for B-form DNA. The relative spatial relationship between these two triads is parameterized by three translational and three rotational misalignments. The misalignments between the B-triads of nodes n_b and n_t, as well as the restorative forces and moments, are calculated in the procedure described by equations (5-8), where the stiffness coefficients are chosen to be $k_{t1} = k_{t2} = k_{t3} = 10^3\ (S_{dsDNA}/L) = 3.24\times10^6$ pN nm^{-1}, $k_{r1} = k_{r2} = 10^3\ (B_{dsDNA}/L) = 6.76\times10^5$ pN nm rad^{-1}, and $k_{r3} = 10^3\ (T_{dsDNA}/L) = 1.35\times10^6$ pN nm rad^{-1}.

Supplementary Note 4 Application to the immobile four-way junction

The present work applies the preceding structure calculation procedure to "stacked-X" four-way junctions that are assumed to exist in their free, ground-state configuration with 60° scissor-like interhelical angle J_{twist} (Supplementary Fig. 6), although the model can equally be applied to anti-parallel junctions as modeled previously in lattice-based origami[2, 3]. At the high magnesium concentrations used in DNA nanostructure self-assembly, isolated four-way junctions are known to exist in solution in two alternative "stacked-X" isomeric states in which duplex arms alter their neighboring stacking interactions[1, 4, 5] (Supplementary Fig. 6). In each of these stacking arrangements, neighboring duplexes adopt a right-handed configuration with J_{twist} that varies from approximately 40° to 60° in the stress-free, mechanical ground-state configuration[6, 7, 8]. Importantly, stacked duplexes are likely to adopt distinct ground-state values of J_{twist} depending on their sequence and the structural context in which they are designed. For example, if duplexes are forced to reside on a lattice in which they adopt an anti-parallel configuration, four-way junctions have been observed to adopt a left-handed configuration with a crossing angle that varies from 10° to 40° [9], although this observation may be attributable to the nature of the specific structure examined in that work. Nevertheless, these experimental observations, together with solution-based FRET measurements, suggest that J_{twist} exhibits significant flexibility and may adopt multiple ground-state configurations depending on the external structural constraints that are imposed on it by adjoined helices. This is similar to RNA junctions that are also known to exhibit highly complex geometrical and mechanical behavior depending on their structural context and solvent environment[10].

To proceed, in the present work four-way junctions are assumed *a priori* to exist in a single "stacked-X" isomeric state with a single ground-state equilibrium angle of 60°. Future work may aim to relax this assumption on isomeric state; determine the conditions under which four-way junctions reside in parallel versus anti-parallel versus non-parallel crossing angles, or in alternative ground-state angles; incorporate nonlinear junction flexibility; and extend the model to other multi-way junctions.

Under this assumption, the immobile four-way junction model consists of two sets of duplex arms with bottom and top helices (BH and TH) chosen arbitrarily when the sequence and topology information as a directed graph is divided into junctions (Supplementary Fig. 1). The termini of arms I and III of the four-way junction are defined as the 5′-end of the non-crossing strands in the BH and TH, respectively. Similarly, the termini of arms II and IV are the 3′-end of the non-crossing strands in the BH and TH, respectively (Supplementary Fig. 7a). Whereas the

ground-state value of J_{twist} is likely to adopt local values neighboring 0° and 180° for anti-parallel and parallel helices, respectively, designed on square or honeycomb lattices, the same model described here may be adapted to these cases, which is explored in separate work.

As an illustrative example, consider application to a four-way junction consisting of two 16-bp-long duplexes (Supplementary Fig. 7a). Each bp is represented as a finite element node labeled as shown in Supplementary Fig. 7a. A reference frame defined by the center position $\mathbf{e}_0$ and three directional vectors $\mathbf{e}_1$, $\mathbf{e}_2$, and $\mathbf{e}_3$ together with the two geometric parameters interhelical distance s and interhelical angle J_{twist} uniquely determines the initial configuration of the finite element model. In this initial configuration, both BH and TH are straight, and the position and orientation of each node can be calculated from s and J_{twist}. For example, the center of the BH, or the midpoint of nodes 8 and 9 at the crossover site in Supplementary Fig. 7a has coordinate $\begin{pmatrix} 0 & 0 & -s/2 \end{pmatrix}$ in the reference frame defined by the center $\mathbf{e}_0$ and axes $\mathbf{e}_1$, $\mathbf{e}_2$, and $\mathbf{e}_3$. Similarly, the midpoint of nodes 24 and 25 at the crossover site in the TH has coordinate $\begin{pmatrix} 0 & 0 & s/2 \end{pmatrix}$ in the reference frame. The vector in the BH from the terminus of arm I (node 1) to that of arm II (node 16) is collinear with $\mathbf{e}_1$ while the vector in the TH from the terminus of arm IV (node 32) to that of arm III (node 17) is collinear with a unit vector obtained by rotating $\mathbf{e}_1$ by J_{twist} about $\mathbf{e}_3$. The finite element nodes are then placed on the BH and TH using the axial rise per bp (L = 0.34 nm) and the right-handed twisting angle per bp (360°/10.5) about the helical axis of B-form DNA.

BH and TH are connected at the crossover site using two individual alignment elements that define the flexibility of the junction. One alignment element connects nodes 8 and 25 while the other one connects nodes 9 and 24 independently. For each alignment element, a triad with its center position $\mathbf{b}_{b0}$ and three directional vectors $\mathbf{b}_{b1}$, $\mathbf{b}_{b2}$, and $\mathbf{b}_{b3}$ is rigidly connected to the node in the BH, and similarly another triad with its center position $\mathbf{b}_{t0}$ and three directional vectors $\mathbf{b}_{t1}$, $\mathbf{b}_{t2}$, and $\mathbf{b}_{t3}$ is rigidly connected to the node in the TH, as described in Supplementary Fig. 7b,c. These two triads coincide with the reference frame at the ground state, i.e. $\mathbf{b}_{bi} = \mathbf{b}_{ti} = \mathbf{e}_i$, i = 0, 1, 2, & 3.

Without loss of generality, the relative rotation between the BH and TH is equivalent to the absolute rotation of the TH while fixing the BH and the reference frame defined by the center $\mathbf{e}_0$ and axes $\mathbf{e}_1$, $\mathbf{e}_2$, and $\mathbf{e}_3$. Thus, three rotational DOFs of the four-way junction are the rotations of the TH about the axes $\mathbf{e}_1$, $\mathbf{e}_2$, and $\mathbf{e}_3$, respectively. Misalignments shown in Supplementary Fig. 7d can be calculated using equations (5-7). Restorative forces and moments are calculated from the misalignments using k_{twist} as the junction twist angle stiffness about axis $\mathbf{e}_3$.

In conclusion, our finite element model for the “stacked-X” four-way junction is defined by the geometric parameters, s and J_{twist}, and the mechanical compliance parameters, k_{t1}, k_{t2}, k_{t3}, k_{r1}, k_{r2}, and k_{twist} (Supplementary Table 1).

Estimation of four-way junction ground-state geometry

The principal geometric parameters defining the “stacked-X” four-way junction are the crossing angle J_{twist} between the duplexes and their inter-axial separation s at the crossover point (Supplementary Fig. 8). There have been various experimental approaches to estimate the ground-state interhelical angle J_{twist} between two sets of coaxially stacked duplex arms in a “stacked-X” four-way junction. For example, FRET has been used to obtain the angle by measuring the distance between chromophores attached to the termini of the four-way junction arms[11]. Time-resolved FRET further has yielded the probability distribution of the angle[12]. Birefringence decay time has also been used to measure the angle in solution[13]. And J_{twist} has been directly measured using a crystal packing model of B-form DNA[14]. Finally, atomic force microscopy (AFM) images of synthesized DNA parallelograms consisting of a lattice of four-way

junctions enable observation of the J_{twist} in the context of a larger-scale self-assembled DNA structure[8]. Each of the preceding approaches has reported a consensus value of approximately 60° for the J_{twist} when the junction resides in a stress-free state. Using the same definition of J_{twist}, atomic structures determined by X-ray crystallography have yielded a somewhat smaller value of J_{twist} which is approximately 40° [6, 7, 15, 16], which does not result from crystal packing[17], but has been attributed to junction sequence, as confirmed by AFM[17]. This sequence-dependence of the four-way junction structure has also been observed in crystallographic studies[18, 19]. While the empirical value of J_{twist} that is assumed here is consistent with observations of unconstrained four-way junctions, application of our model to anti-parallel or parallel DNA helices constrained by high-density crossovers may require values closer to 0° or 180°. Future work will explore the optimal choice of this value using high resolution cryo-electron microscopy (cryo-EM) data from origami[20] and cage-like objects[21, 22, 23].

The junction geometry also depends on the interhelical distance s. The cryo-EM structure of a megadalton-scale DNA origami suggests an interhelical distance of s = 1.85 nm[20], which is consistent with the value of crystal structures of Holliday junctions[7]. Importantly, interhelical repulsion between neighboring duplexes due to electrostatic interactions which are not modeled here, may lead to a somewhat larger effective interhelical spacing of 2.2 nm, as assumed previously[2, 3]. Nevertheless, we chose to assume s = 1.85 nm to be consistent with known atomic structures of the junction.

Estimation of four-way junction ground-state mechanical properties

Our core assumption is that four-way junctions are principally compliant in twisting of the relative scissor-like twisting angle between the stacked duplexes, namely J_{twist}. Four-way junctions are therefore assumed to be effectively rigid in their relative three translational DOFs, $k_{t1} = k_{t2} = k_{t3} = 2\times10^3 (S_{dsDNA}/L) = 6.47\times10^6$ pN nm^{-1}. This assumption is justified by the topological constraint imposed by the connectivity of crossovers of continuous DNA strands. We further assume that each of the two alignment elements (Supplementary Fig. 7b,c) have the same bending stiffness as B-form DNA and thus set two rotational stiffness coefficients of the four-way junction to $k_{r1} = k_{r2} = 2(B_{dsDNA}/L)$ = 1,353 pN nm rad^{-1}. The junction twisting stiffness, k_{twist}, is estimated below from MD simulations and verified using published FRET data.

Four independent 100-ns-long MD simulation replicates were performed using the crystal structure of the four-way junction PDB ID 1DCW[7]. Explicit Mg^{2+}, Na^{+}, and Cl^{-} ions were fitted to the DNA electrostatic potential in the absence of water using the CIonize program[24] to produce a zero net charge and salt concentrations of 18 mM $MgCl_2$ and 5 mM NaCl, consistent with experimental conditions[25]; followed by immersion in a periodic cubic box of explicit TIP3P water. Simulations were performed in the isothermal-isobaric ensemble at 300 K and 1 atm using the software NAMD[26] and the CHARMM27 parameter set[27, 28] with modified Mg^{2+} parameters[29]. The modified magnesium parameters were calibrated to the CHARMM27 field, hence its use in preference to the CHARMM36 field. Temperature and pressure were maintained using the Langevin dynamics and Langevin piston formalisms according to the protocol used for explicit simulation of 1DCW[30]. A 2 fs time-step was used with all hydrogens constrained to their equilibrium lengths using the SHAKE and RATTLE algorithms. A shifted cut-off from 10 to 12 Å was used, with long-range electrostatics represented by a Particle Mesh Ewald summation. 5,000 steps of energy minimization were performed with the conjugate-gradient line minimizer, followed by controlled heating and equilibration over 6 ns before final production runs of 100 ns each. The same force field, ion and solvent models were used for the minimization, equilibration, and final production runs.

Junction conformations were stored every ps from 20 ns to 100 ns in each MD simulation. Thus, 320,000 conformations were sampled in total from the four MD simulations (Supplementary Fig. 9a). Note that the MD simulations only sample conformations with J_{twist}

close to its ground-state value (Supplementary Fig. 9a). A J_{twist} was computed for each sampled conformation, and the variance $\mathrm{var}(J_{twist})$ = 0.027 rad^2 among the 320,000 conformations (Supplementary Fig. 9b). The equipartition theorem yields the rotational stiffness k_{twist} = $k_B T/\mathrm{var}(J_{twist}) \approx 0.1(T_{dsDNA}/L)$ = 135.3 pN nm rad^{-1}, where k_B is the Boltzmann constant.

In order to perform an independent test of the estimated value of k_{twist} from MD simulations, we simulated single-molecule FRET (smFRET) measurements on Cy3 and Cy5 dyes tethered to the ends of two arms of a free "stacked-X" four way junction in a specific isomeric state consisting of 11 bps in each arm using the parameterized finite element model of the four-way junction (Supplementary Fig. 10a)[1].

Experimentally measured FRET efficiencies E in the junction follow a Gaussian distribution with probability density,

$$p(E) = \frac{1}{\sigma_E\sqrt{2\pi}}\exp\left[-\frac{(E-\mu_E)^2}{2\sigma_E^{\ 2}}\right] \tag{9}$$

where the mean μ_E = 0.51 and the standard deviation σ_E = 0.11[1].

FRET efficiencies were calculated in six independent simulations in which the standard value of the rotational stiffness, k_{twist} = 135.3 pN nm rad^{-1}, was multiplied by factors a = 0.2, 1, 2, 20, 200, and 2000, respectively. In each simulation, we first generated the atomic structure of the junction and then, a trajectory of 1,000 conformations was generated by performing finite element normal mode analysis[31]. The two accessible volumes (AVs)[32, 33] of the Cy3 and Cy5 tethered dyes were generated in each conformation (Supplementary Fig. 10a). The mean FRET efficiency in a given conformation was then calculated using static averaging[31],

$$E = \left\langle 1 \middle/ \left[1 + \frac{2}{3\kappa^2}\left(\frac{R}{R_0}\right)^6\right] \right\rangle \tag{10}$$

Where R is the distance between two points $\mathbf{x}_{Cy3}$ and $\mathbf{x}_{Cy5}$ randomly chosen from the AVs of Cy3 and Cy5, respectively, in the current conformation. The Förster radius between Cy3 and Cy5 is R_0 = 5.4 nm[34, 35] when the orientation factor κ^2 = 2/3. The actual orientation factor considering rotation of the dyes was given by,

$$\kappa = \mathbf{e}_1 \cdot \mathbf{e}_2 - 3(\mathbf{e}_1 \cdot \mathbf{e}_{12})(\mathbf{e}_{12} \cdot \mathbf{e}_2) \tag{11}$$

where $\mathbf{e}_1$ and $\mathbf{e}_2$ are two random unit vectors, and $\mathbf{e}_{12} = (\mathbf{x}_{Cy5} - \mathbf{x}_{Cy3})/R$. Thus, 1,000 mean FRET efficiencies were obtained in each simulation.

For each simulation, the distribution of the FRET efficiencies E calculated with equation (10) was compared to that measured in experiment (Supplementary Fig. 10b). The distributions of simulated FRET efficiencies with a = 1, 2, 20, 200, and 2000 are all insensitive to the value of a and all agree with the experiment. In contrast, the simulated FRET efficiencies with a = 0.2 follow a flat and broad distribution, differing significantly from experiment. These results suggest a lower bound of 0.2 k_{twist} on the rotational stiffness coefficient, justifying the value found from fluctuation analysis in the preceding MD simulations.

Supplementary References

1. Hyeon C, Lee J, Yoon J, Hohng S, Thirumalai D. Hidden complexity in the isomerization dynamics of Holliday junctions. *Nat Chem* **4**, 907-914 (2012).
2. Castro CE*, et al.* A primer to scaffolded DNA origami. *Nat Methods* **8**, 221-229 (2011).
3. Kim DN, Kilchherr F, Dietz H, Bathe M. Quantitative prediction of 3D solution shape and flexibility of nucleic acid nanostructures. *Nucleic Acids Res* **40**, 2862-2868 (2012).
4. Lilley DMJ. Structures of helical junctions in nucleic acids. *Q Rev Biophys* **33**, 109-159 (2000).
5. McKinney SA, Declais AC, Lilley DMJ, Ha T. Structural dynamics of individual Holliday junctions. *Nat Struct Biol* **10**, 93-97 (2003).
6. Ortiz-Lombardia M, Gonzalez A, Eritja R, Aymami J, Azorin F, Coll M. Crystal structure of a DNA Holliday junction. *Nat Struct Biol* **6**, 913-917 (1999).
7. Eichman BF, Vargason JM, Mooers BHM, Ho PS. The Holliday junction in an inverted repeat DNA sequence: Sequence effects on the structure of four-way junctions. *Proc Natl Acad Sci USA* **97**, 3971-3976 (2000).
8. Mao CD, Sun WQ, Seeman NC. Designed two-dimensional DNA Holliday junction arrays visualized by atomic force microscopy. *J Am Chem Soc* **121**, 5437-5443 (1999).
9. Yoo J, Aksimentiev A. In situ structure and dynamics of DNA origami determined through molecular dynamics simulations. *Proc Natl Acad Sci USA* **110**, 20099-20104 (2013).
10. Bindewald E, Hayes R, Yingling YG, Kasprzak W, Shapiro BA. RNAJunction: a database of RNA junctions and kissing loops for three-dimensional structural analysis and nanodesign. *Nucleic Acids Res* **36**, D392-D397 (2008).
11. Murchie AIH, Clegg RM, von Kitzing E, Duckett DR, Diekmann S, Lilley DMJ. Fluorescence energy transfer shows that the four-way DNA junction is a right-handed cross of antiparallel molecules. *Nature* **341**, 763-766 (1989).
12. Eis PS, Millar DP. Conformational distributions of a four-way DNA junction revealed by time-resolved fluorescence resonance energy transfer. *Biochemistry* **32**, 13852-13860 (1993).
13. Cooper JP, Hagerman PJ. Geometry of a branched DNA structure in solution. *Proc Natl Acad Sci USA* **86**, 7336-7340 (1989).
14. Timsit Y, Moras D. Groove-backbone interaction in B-DNA implication for DNA condensation and recombination. *J Mol Biol* **221**, 919-940 (1991).
15. Vargason JM, Ho PS. The effect of cytosine methylation on the structure and geometry of the Holliday junction. *J Biol Chem* **277**, 21041-21049 (2002).
16. Watson J, Hays FA, Ho PS. Definitions and analysis of DNA Holliday junction geometry. *Nucleic Acids Res* **32**, 3017-3027 (2004).
17. Sha RJ, Liu FR, Seeman NC. Force microscopic measurement of the interdomain angle in symmetric Holliday junctions. *Biochemistry* **41**, 5950-5955 (2002).
18. Hays FA*, et al.* How sequence defines structure: A crystallographic map of DNA structure and conformation. *Proc Natl Acad Sci USA* **102**, 7157-7162 (2005).
19. Hays FA, Vargason JM, Ho PS. Effect of sequence on the conformation of DNA Holliday junctions. *Biochemistry* **42**, 9586-9597 (2003).
20. Bai XC, Martin TG, Scheres SHW, Dietz H. Cryo-EM structure of a 3D DNA-origami object. *Proc Natl Acad Sci USA* **109**, 20012-20017 (2012).
21. He Y*, et al.* Hierarchical self-assembly of DNA into symmetric supramolecular polyhedra. *Nature* **452**, 198-U141 (2008).
22. Bhatia D, Mehtab S, Krishnan R, Indi SS, Basu A, Krishnan Y. Icosahedral DNA nanocapsules by modular assembly. *Angew Chem Int Edit* **48**, 4134-4137 (2009).
23. Seeman NC. Nanomaterials based on DNA. *Annu Rev Biochem* **79**, 65-87 (2010).

24. Stone JE, Phillips JC, Freddolino PL, Hardy DJ, Trabuco LG, Schulten K. Accelerating molecular modeling applications with graphics processors. *J Comput Chem* **28**, 2618-2640 (2007).
25. Dietz H, Douglas SM, Shih WM. Folding DNA into twisted and curved nanoscale shapes. *Science* **325**, 725-730 (2009).
26. Phillips JC*, et al.* Scalable molecular dynamics with NAMD. *J Comput Chem* **26**, 1781-1802 (2005).
27. Foloppe N, MacKerell AD. All-atom empirical force field for nucleic acids: I. Parameter optimization based on small molecule and condensed phase macromolecular target data. *J Comput Chem* **21**, 86-104 (2000).
28. MacKerell AD, Banavali NK. All-atom empirical force field for nucleic acids: II. Application to molecular dynamics simulations of DNA and RNA in solution. *J Comput Chem* **21**, 105-120 (2000).
29. Allner O, Nilsson L, Villa A. Magnesium ion-water coordination and exchange in biomolecular simulations. *J Chem Theory Comput* **8**, 1493-1502 (2012).
30. Yu J, Ha TJ, Schulten K. Conformational model of the Holliday junction transition deduced from molecular dynamics simulations. *Nucleic Acids Res* **32**, 6683-6695 (2004).
31. Pan K, Boulais E, Yang L, Bathe M. Structure-based model for light-harvesting properties of nucleic acid nanostructures. *Nucleic Acids Res* **42**, 2159-2170 (2014).
32. Kalinin S*, et al.* A toolkit and benchmark study for FRET-restrained high-precision structural modeling. *Nat Methods* **9**, 1218-U1129 (2012).
33. Sindbert S*, et al.* Accurate distance determination of nucleic acids via Förster resonance energy transfer: implications of dye linker length and rigidity. *J Am Chem Soc* **133**, 2463-2480 (2011).
34. Ha T*, et al.* Initiation and re-initiation of DNA unwinding by the Escherichia coli Rep helicase. *Nature* **419**, 638-641 (2002).
35. Yuan FQ, Griffin L, Phelps LJ, Buschmann V, Weston K, Greenbaum NL. Use of a novel Förster resonance energy transfer method to identify locations of site-bound metal ions in the U2-U6 snRNA complex. *Nucleic Acids Res* **35**, 2833-2845 (2007).
