# Supplementary material for: Lattice-free prediction of three-dimensional structure of programmed DNA assemblies
Source: Nat Commun. 2014 Dec 3;5:5578. doi: 10.1038/ncomms6578 (PMC4268701; doi:10.1038/ncomms6578)
Supplement: Supplementary Dataset 1 — Tiamat and PDB files for 3D crystal lattice. [file ncomms6578-s10.zip › Supplemetary Dataset 1/Filenames.pdf]

**DNA Nanostructure**	**Tiamat filename**	**PDB filename**
4-layer ring	4_layer.dna	4_layer.pdb
9-layer ring origami	9_layer.dna	9_layer.pdb
12-layer hemispherical origami	12_layer.dna	12_layer.pdb
40×2 ribbon with [n_x, n_y] = [21, 21] bps	21_21_40x2.dna	21_21_40x2.pdb
40×4 ribbon with [n_x, n_y] = [21, 21] bps	21_21_40x4.dna	21_21_40x4.pdb
40×2 ribbon with [n_x, n_y] = [22, 21] bps	22_21_40x2.dna	22_21_40x2.pdb
40×4 ribbon with [n_x, n_y] = [22, 21] bps	22_21_40x4.dna	22_21_40x4.pdb
40×2 ribbon with [n_x, n_y] = [20, 21] bps	20_21_40x2.dna	20_21_40x2.pdb
40×4 ribbon with [n_x, n_y] = [20, 21] bps	20_21_40x4.dna	20_21_40x4.pdb
40×2 ribbon with [n_x, n_y] = [22, 22] bps	22_22_40x2.dna	22_22_40x2.pdb
40×4 ribbon with [n_x, n_y] = [22, 22] bps	22_22_40x4.dna	22_22_40x4.pdb
40×2 ribbon with [n_x, n_y] = [20, 20] bps	20_20_40x2.dna	20_20_40x2.pdb
40×4 ribbon with [n_x, n_y] = [20, 20] bps	20_20_40x4.dna	20_20_40x4.pdb
3D crystal lattice	3D_crystal.dna	3D_crystal.pdb
